# New Insights in Thrombin Inhibition Structure–Activity Relationships by Characterization of Octadecasaccharides from Low Molecular Weight Heparin †

**DOI:** 10.3390/molecules22030428

**Published:** 2017-03-08

**Authors:** Pierre A. J. Mourier, Olivier Y. Guichard, Fréderic Herman, Philippe Sizun, Christian Viskov

**Affiliations:** Sanofi, 13 Quai Jules Guesde, 94403 Vitry sur Seine, France; pierre.mourier@sanofi.com (P.A.J.M.); olivier.guichard@sanofi.com (O.Y.G.); frederic.herman@sanofi.com (F.H.); philippe.sizun@sanofi.com (P.S.)

**Keywords:** thrombin inhibition, LMWH, antithrombin, heparin oligosaccharides, ternary complex

## Abstract

Low Molecular Weight Heparins (LMWH) are complex anticoagulant drugs that mainly inhibit the blood coagulation cascade through indirect interaction with antithrombin. While inhibition of the factor Xa is well described, little is known about the polysaccharide structure inhibiting thrombin. In fact, a minimal chain length of 18 saccharides units, including an antithrombin (AT) binding pentasaccharide, is mandatory to form the active ternary complex for LMWH obtained by alkaline β-elimination (e.g., enoxaparin). However, the relationship between structure of octadecasaccharides and their thrombin inhibition has not been yet assessed on natural compounds due to technical hurdles to isolate sufficiently pure material. We report the preparation of five octadecasaccharides by using orthogonal separation methods including size exclusion, AT affinity, ion pairing and strong anion exchange chromatography. Each of these octadecasaccharides possesses two AT binding pentasaccharide sequences located at various positions. After structural elucidation using enzymatic sequencing and NMR, in vitro aFXa and aFIIa were determined. The biological activities reveal the critical role of each pentasaccharide sequence position within the octadecasaccharides and structural requirements to inhibit thrombin. Significant differences in potency, such as the twenty-fold magnitude difference observed between two regioisomers, further highlights the importance of depolymerisation process conditions on LMWH biological activity.

## 1. Introduction

Low Molecular Weight Heparins (LMWH) are lifesaving anticoagulant drugs which have been used for several decades in the prevention and treatment of venous and arterial thromboembolism [1]. LMWH are industrially derived from the starting material heparin, a complex mixture of mammalian polysaccharides extracted from porcine mucosa. The centenary of the discovery of heparin has been widely celebrated around the world and many symposia, such as the 24th Glycosaminoglycans Symposium, held in September 2016 (Villa Vigoni, Loveno di Menaggio—Italy, the first symposia of these series was initiated by Pr Casu), were dedicated to this endeavor. Even with state of the art analytical methods, this “ever young life-saving drug” [2] is also one of the most complex products to analyze, as direct characterization of the polysaccharidic chains is still not feasible. Heparin is a mixture of heteropolymeric chains having a mean molecular weight around 15,000 Dalton. It consists of alternating units of 2-deoxy-2-sulfamido-α-d-glucopyranose (possibly *O*-sulfated, *N*-sulfated or *N*-acetylated) and *O*-sulfated uronic acids (α-l-iduronic acid or β-d-glucuronic acid). The specific structure of each polysaccharidic chain, built up of these alternating motifs, is governed by enzymatic machinery of the host mast cells where it is stored [3]. Heparin interacts with about one hundred proteins [4] but its anticoagulant activity is mainly mediated indirectly through activation of antithrombin, a serine protease inhibitor, member of the serpin family [5]. The activation of antithrombin induces a protein conformational change and dramatically accelerates the inhibition of the Stuart factor (FXa) [6]. The discovery of the antithrombin (AT) binding pentasaccharide was the hallmark of a new era of understanding of the structure activity of this complex medicine [7,8], and the so-called AGA*IA sequence (GlcN_NAc/NS,6S_-GlcA-GlcN_NS,3S,6S_-IdoUA_2S_GlcN_NS,6S_) was identified as the specific binding sequence of heparin [7]. Then, several variants of the consensus pentasaccharide sequence were discovered and reported a posteriori [9,10,11]. The evolution of analytical methodologies has also permitted the identification of longer AT binding oligosaccharides from LMWH [12]. Structure-activity studies on octa to octadecasaccharides have demonstrated that interaction with antithrombin is more complex than initially envisioned and that flanking saccharide units play an important role in the modulation of the AGA*IA affinity (the synthetic version of AGA*IA, drug substance commercially known as fondaparinux was used as reference) [13]. They may affect the Kd variation by three orders of magnitude when compared to fondaparinux (Kd = 21 nM) [14], either strengthening or destabilizing this complex.

Furthermore, semuloparin an experimental drug for which development was stopped in 2012 was designed with a depolymerisation process which preserves the AT binding region from β-eliminative cleavage and increases their concentration in this particular LMWH [15]. Therefore, it allowed the isolation of unexpected multiple of two and three consecutive AGA*IA sequences in the dodecasaccharide ΔIIa-IIs-Is-IIa-IIs-Is [16] and the octadecasaccharide **1** ΔIIa-IIs-Is-IIa-IIs-Is-IIa-IIs-Is [17], respectively (structural symbols are listed in Table 1; previous studies had concluded the possible presence of two AT binding sites on a single heparin chain without isolation and structural elucidation [18]). This compositional specificity is directly due to the depolymerisation selectivity with the sterically hindered phosphazene bases. Therefore, semuloparin appears as a particularly interesting tool compound to gather new insights on structure-activity relationships.

It is noteworthy that we have previously observed that Kd affinity and molar anti FXa activity of the dodecasaccharide was twice that of fondaparinux, suggesting a dynamic equilibrium with two antithrombin proteins, which may potentiate the anticoagulant activity of such compound series. 

**Figure 1:**
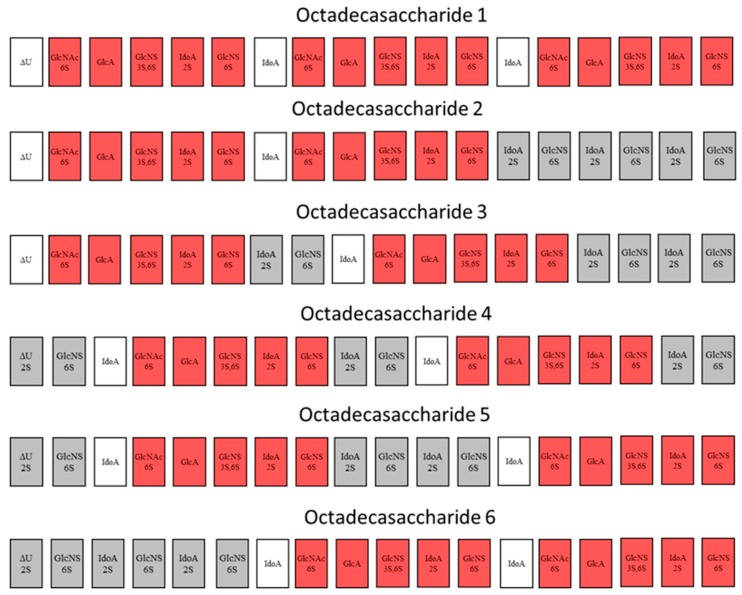
Studied octadecasaccharides (in red saccharide units from AGA*IA sequence). In grey, flanking saccharide units are represented. In white, additional uronic acid present in the minimal natural sequence.

Although thrombin is one of the key coagulation proteases for heparin and LMWHs, structure-activity data are not available due to the challenge of isolating polysaccharides that are able to form the ternary complex with antithrombin and thrombin. In fact, until the isolation of octadecasaccharide **1** (Figure 1) [17], there was no structural data on pure natural compounds that were responsible for this important mechanism of action. Interestingly, the only structure-activity relationship evidence that existed were generated on synthetic compounds [19,20,21] and showed that the inhibition may be potent starting from hexadecasaccharides.

With the octadecasaccharide **1**, a suitable chain length was reached so that the inhibition of the thrombin (FIIa) became possible in the case of LMWH products (no significant activity assayed on hexadecasaccharides), and activities of aFXa = 562 IU/mg and aFIIa = 8.8 IU/mg were found. Nevertheless, with respect to the whole fraction from which it was isolated (aFIIa ~20 UI/mg), the aFIIa for 1 was significantly lower than expected. 

It was therefore critical to further evaluate new octadecasaccharides to increase our understanding of the electrostatic interactions with thrombin and compare them to the synthetic compounds from Petitou et al. [19,20,21].

For this endeavor, by using orthogonal chromatographic techniques, we have isolated five compounds from the octadecasaccharide AT affine fraction of semuloparin. Each of these compounds contain two AGA*IA sequences located at different positions within the polysaccharide chain (Figure 1). Their structure has been fully elucidated by enzymatic sequencing and NMR analysis and their anticoagulant activity was evaluated both for inhibition of factor Xa and IIa.

## 2. Results and Discussion

In our previous work [17], we reported the isolation from semuloparin of the first natural octadecasaccharide having AT binding affinity and able to inhibit both aFXa and aFIIa. This compound had the peculiarity to bear three consecutive AGA*IA sequences. Surprisingly, the activation of thrombin was weak (only 8.8 IU/mg; USP Heparin is about 180 IU/mg) despite the fact that it was isolated from an octadecasaccharide fraction with a specific aFIIa activity of ~20 UI/mg. All the criteria for activity were there, i.e., size of the chain, presence of AGA*IA sequences, and high anti FXa (about three times more than USP heparin) activity, but the low thrombin inhibition raised new questions. Front running hypotheses to explain low thrombin inhibition included: a lack of charge density to stabilize the ternary complex with the protein, or a dynamic equilibrium between antithrombin with all three AGA*IA sequences, which might generate in turn a steric hindrance for thrombin complexation. The later hypothesis could also explain the twofold molar aFXa activity of the compound with respect to the pentasaccharide [17]. To shed light on the origin of such biological properties, it was critical to study more compounds of these series and put in perspective the work performed on synthetic polysaccharides with dual anti FXa and thrombin inhibition potency [19,21].

### 2.1. Purification of Octadecasaccharide Fraction F3

**Figure 2:**
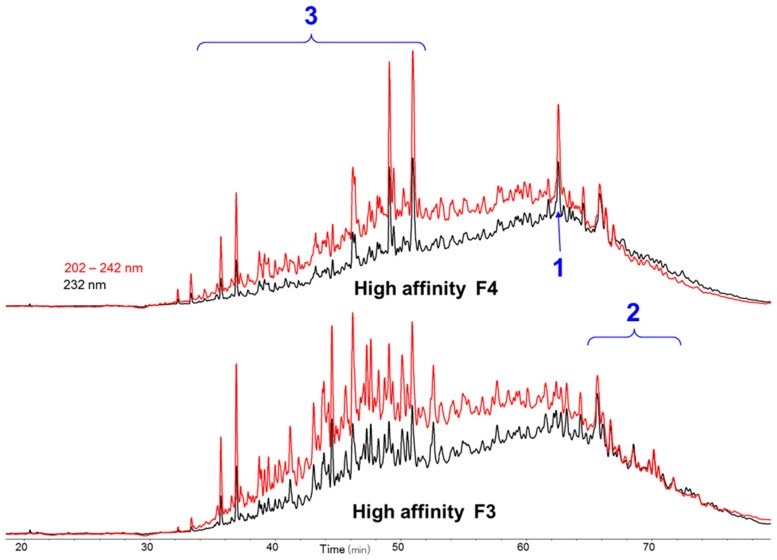
Cetyltrimethylammonium strong anion exchange (CTA-SAX) chromatograms of octadecasaccharide fractions F3 and F4: 1-triple site, 2-double sites, 3-glycoserine octadecasaccharides.

The octadecasaccharide fraction was separated on an AT affinity chromatography column and five fractions F1 to F5 with increasing affinity were isolated [17]. Focus was concentrated on fraction F3, where isomers containing two AGA*IA sequences could be identified (Figure 2).

**Figure 3:**
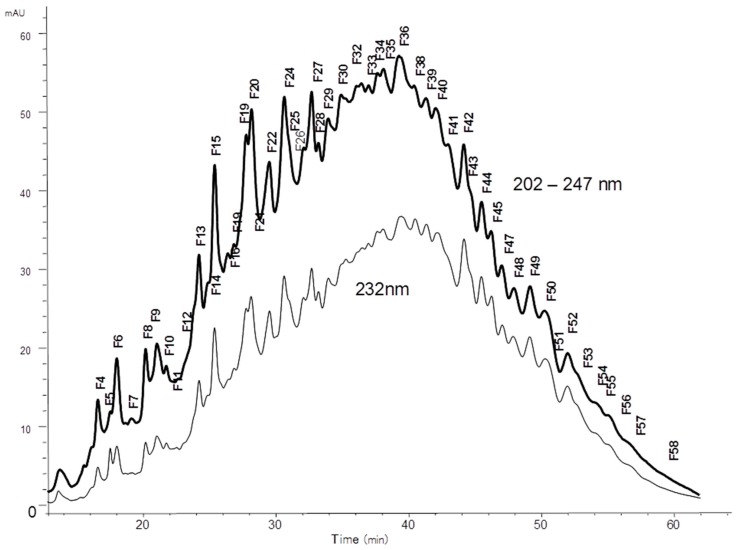
Preparative CTA-SAX chromatography of fraction F3.

**Figure 4:**
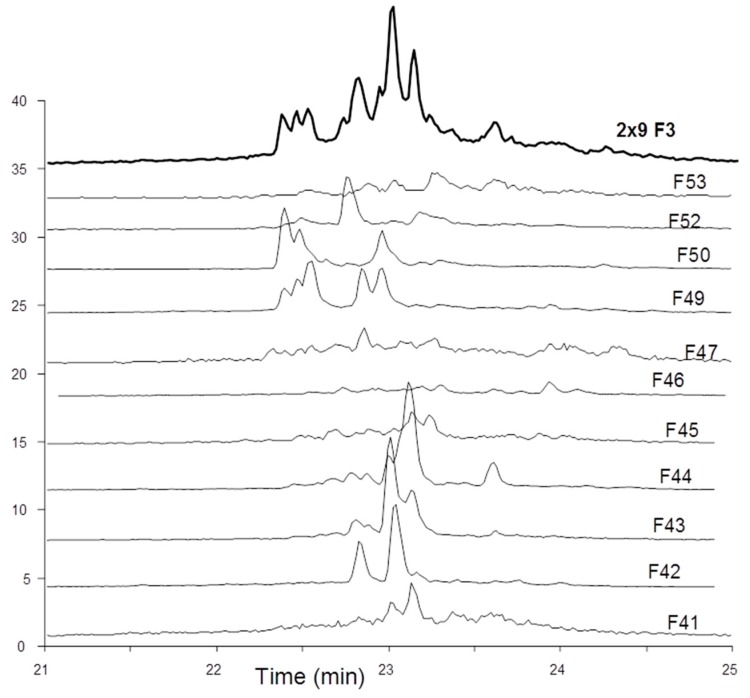
LC/MS reconstructed ion mass chromatograms of initial fraction F3 and sub fractions of interest corresponding to MW 4957 Da (*m*/*z* 2430.2: (4957 + 23 × HXA + 2H)^3+^).

The overall activity was in the same order of magnitude than fraction 4 from which octadecasaccharide **1** was isolated (aFXa: F3 401 IU/mg; F4 422 IU/mg; aFIIa: F3 18.1 IU/mg; F4 19.8 IU/mg). The modest aFIIa activity may be partly related to the presence of glycoserine oligosaccharides. These oligosaccharides contain an oxidized glycoserine sequence (-GlcA-Gal-Gal-Xyl-CH_2_-COOH) at their reducing end [22]. These compounds are eluted in the less retained part of the chromatogram (Figure 2) and represent more than 50% of the affine fractions. None of the glycoserine octadecasaccharides isolated in this study (not described here) had any measurable aFIIa activity while having aFXa. Compared to the separation of the octadecasaccharide **1**, the isolation of five regioisomers containing two AGA*IA sequences was more complex and the resolution power of ion pair chromatography was critical to enable the separation. From the key step of cetyltrimethylammonium strong anion exchange (CTA-SAX) separation (Figure 3), reconstructed ion mass LC/MS chromatograms corresponding to Mw 4957 Da (Figure 4) were used to select the fractions to be gathered. Molecular weights deduced from LC/MS analysis were sufficient to deduce the sulfate group number, *N*-acetyl number and presence or not of characteristic glycoserine moieties. These pieces of information were helpful to guide us in the last purification steps.

### 2.2. Enzymatic Sequencing

Sequencing methods based on partial or selective depolymerisation with enzymes from *Flavobacterium heparinum* and particularly heparinases have been already developed [23,24]. Monitoring of digestion and fragment analysis were done by MALDI-MS and capillary electrophoresis. Another efficient technique using a preliminary radiolabeling of the substrate, followed by partial nitrous depolymerisation and sequencing with exoenzymes has been proposed [25]. However, this method is long and most exoenzymes used in the study are not commercially available. In fact, the key element for any sequencing method is an efficient separation. This part is usually time consuming and, as already stated, requires use of many consecutive chromatographic methods with orthogonal specificities.

For long heparin-like oligosaccharides, heparinase I is the most convenient choice for sequencing experiments. Heparinase II is less selective than heparinase I and, as an exolithic enzyme, it does not generate many long fragments in detectable amounts. The full exploitation of the digestion process by heparinase I requires a good understanding of the enzyme’s selectivity and behavior. Basic rules for selectivity [26] are necessary but are not sufficient to this endeavor. Know-how in that field is all the more important as current literature data may give only partially the heparinase I mechanism of digestion [27]. As a matter of fact, much evidence exists that heparinase I is not exolithic but rather endolithic [28] (also reflected in our sequencing experiments), all cleavable sites present in the chain being cleaved simultaneously. The structural determination by enzymatic sequencing of long oligosaccharides is usually a challenging experiment due to the number of saccharides bound together and to their potential arrangement. The fact that there are two AT binding sites –IIa_id_-IIs_glu_-Is_id_- distributed with three –Is_id_- disaccharides in an octadecasaccharide simplifies its structural elucidation. Consequently, only 10 theoretical arrangements can be found (Table 2). In addition, the UV spectrum can be used [17] to determine the beginning of the chain (either ΔIIa-IIs_glu_-Is_id_- or ΔIs-), allowing the separation into two types of octadecasaccharides.

#### 2.2.1. Type I Octadecasaccharides

In this case of Type I octadecasaccharides, the position of the first binding site is known and the remaining question is the position of the second one. Key elements for such sequencing were described [17] for structural determination of triple site ΔIIa-IIs_glu_-Is_id_-IIa_id_-IIs_glu_-Is_id_-IIa_id_-IIs_glu_-Is_id_. NaBH_4_ preliminary reduction was used to label the reducing end.

Putative structures are listed in Table 2 as well as the heparinase I cleavage sites of the four possible isomers. Heparinase I cleaves preferentially highly sulfated moieties like -IIs_glu_${}^{\underline{↓}}$Is_id_ and Is_id_${}^{\underline{↓}}$Is_id_-Is_id_^red^ and its action on other cleavable sites like Is_id_${}^{\underline{↓}}$Is_id_^red^ is less pronounced. 

Table 3 gathers key fragments for type I octadecasaccharides. The resistance of these fragments to the action of heparinase I mainly depends on the number of cleaving sites, so that the order of increasing resistance is the following: ΔIIa-IIs_glu_-Is_id_-Is_id_-Is_id_-IIa_id_-IIs_glu_ < ΔIIa-IIs_glu_-Is_id_-Is_id_-IIa_id_-IIs_glu_ < ΔIIa-IIs_glu_-Is_id_-IIa_id_-IIs_glu_ << ΔIs-IIa_id_-IIs_glu_-Is_id_^red^. The sequencing details for octadecasaccharide **2** and **3** are reported in supplementary data.

#### 2.2.2. Type II Octadecasaccharides

The sequencing of type II octadecasaccharides is more challenging than that of type I. In this case, the position of the two binding sites has to be determined. Preliminary 2-O desulfatation of unsaturated acid by Δ^4,5^-glucuronate-2-sulfatase is crucial to differentiate the first AT binding site from the second one. This reaction is achieved before NaBH_4_ reduction and heparinase I addition. It appears that the activity of the sulfatase is highly dependent on the structure and more especially on the number of disaccharide Is at the non-reducing end. Octadecasaccharides **4** and **5** are entirely transformed after one addition of sulfatase. For compound **6**, the desulfatation is very slow, and the quantity of sulfatase necessary to the transformation exceeds all other cases. The activity of *O*-sulfatase is found to be dependent on the substrate structure. The following order is observed: ΔIs-IIa_id_-IIs_glu_- > ΔIs-Is_id_-IIa_id_-IIs_glu_- > ΔIs-Is_id_-Is_id_-IIa_id_-IIs_glu_-. This enzyme has already been cloned and its selectivity studied [29,30] but the selectivity observed above was not heretofore mentioned. Table 3 gathers key fragments generated subsequently after heparinase I treatment for type II octadecasaccharides. Full details of sequencing experiments of octadecasaccharides **4**, **5** and **6** are described in the Supplementary Data. 

### 2.3. NMR

**Figure 5:**
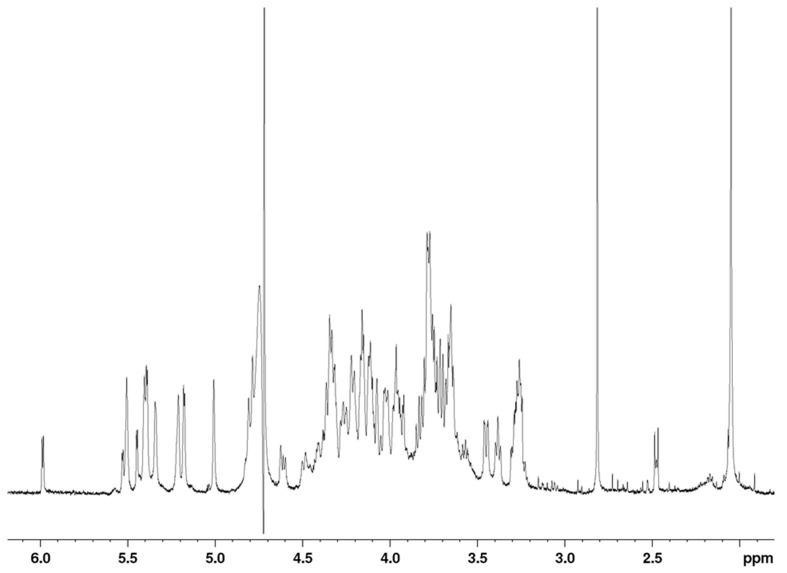
^1^H-NMR spectrum of octadecasaccharide **6** (D_2_O, 30 °C, 600 MHz).

The ^1^H-NMR spectrum of the octasaccharide **6** is shown in Figure 5. Proton and carbon resonances of each residue were assigned using COSY (correlation spectroscopy), TOCSY (total correlation spectroscopy) and HSQC (heteronuclear single quantum coherence spectroscopy) pulse sequences (Table 4) (octadecasaccharides **2**, **3**, **4** and **5** structural assignments are reported in the Supplementary Data).

**Figure 6:**
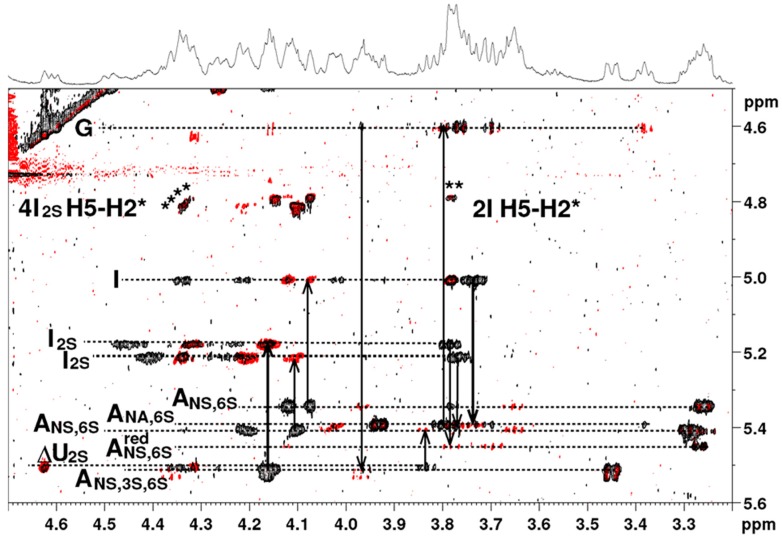
Superimposition of 2D nuclear Overhauser spectroscopy (NOESY) (black) and total correlation spectroscopy (TOCSY) (red) spectra for octadecasaccharide **6**: Correlations of anomeric protons. The dark lines show at start C_i_-H1 to C_(i+1)_-H4 connectivities on the NOESY experiment and at the arrows the C_i_-H1 to C_i_-H4 connectivities on the TOCSY experiment. The H5-H2 characteristic connectivity of ^2^S_0_ conformation of each Iduronic acids is indicated by (*) on the NOESY experiment.

A total of 18 proton signals were observed in the anomeric region between 5.55 and 4.55 ppm, indicating that the polysaccharide is an octadecasaccharide, with only 13 different chemical shifts. As a consequence, the proton spectrum did not appear as complex as might be expected for an octadecasaccharide. This feature indicated the presence of two repeating units in the sequence of the octadecasaccharide, with many resonance superimpositions. Chemical shift analysis was compatible with a composition including nine α-d-glucosamines, six α-l-iduronic acids, two β-d-glucuronic acids (anomeric proton resonance observed between 5.53 and 5,34 ppm, 5.22 and 5.01 ppm and at 4.60 ppm, respectively), and one 4,5-unsaturated uronic acid. Two central acetyl signals are visible at 2.05 ppm. The C_i_-H1 to C_(i+1)_-H4 connectivity, observed in the nuclear Overhauser spectroscopy (NOESY) experiment, were used for monosaccharides sequence determination (Figure 6). The well-resolved resonance of H4-ΔU uronic acid was used as the assignment starting point. The presence of *N*-sulfate or *N*-acetyl on α-d-glucosamine residues, and the presence or absence of *O*-sulfate groups, was deduced from ^1^H and ^13^C chemical shifts.

In spite of the ^1^H signal broadness and many resonance superimpositions, it was possible to determine the main coupling constant for vicinal protons of pyranose rings in the well resolved COSY experiment. For glucosamines and glucuronic acids all ^3^*J*_H1-H2_ coupling constants were superior to 8 Hz, except for the ^3^*J*_H1-H2_ of glucosamines between 3and 4 Hz. Consequently, the conformational status of glucosamines and glucuronic acids was in the pure ^4^C_1_ conformation. All iduronic acids signals showed small coupling constant for vicinal protons, except for the ^3^*J*_H2-H3_ around 7 to 8 Hz. Besides weak-medium intensities were observed for the H5-H2 connectivities in the NOESY experiment (Figure 6). This information taken together suggested a ^1^C_4_↔^2^S0 equilibrium for all iduronic acids [31].

The full proton and carbon chemical shift assignments, along with all sets of inter-residue correlations observed on the 2D NOESY spectrum, fully supported the structural determination of octadecasaccharide **6** with two pentasaccharide sequences as demonstrated by the enzymatic sequencing methodology. The two AGA*IA sequences are respectively located in the middle and at the reducing end of the octadecasaccharide. The NMR assignment tables for the octadecasaccharides **2**, **3**, **4** and **5** are reported in the Supplementary Data.

### 2.4. aFXa and aFIIa Activities

**Figure 7:**
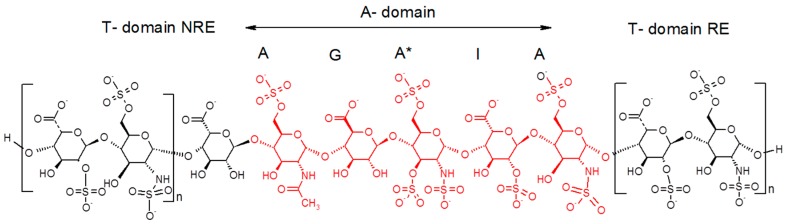
Model described for heparin oligosaccharides with Is disaccharide T domain extensions at reducing end (RE) and non-reducing end (NRE).

In their previous work [19,21], Petitou et al. introduced the concepts of A domain (AT binding domain AGA*IA) and thrombin binding domain (T domain) for their synthetic heparin mimetics. At that time, pure natural structures were not available and therefore, hypotheses had to be made to figure out the AGA*IA flanking oligosaccharides, which might constitute the T domain. Their structure-activity studies showed that the T domain should be highly sulfated in order to interact with the positively charged domain of thrombin [19]. Because heparin has some highly sulfated regular regions with repetitive units of trisulfated Is disaccharides, it was therefore presumed that the T domain was an oligomer of this structural motif (Figure 7).

As Petitou et al. prepared modified simplified structures of both the A and T domain to generate biological data, the results are not directly comparable with those of the natural octadecasaccharide series but at least they provide us some directions. Briefly, in these synthetic oligosaccharides, *N*-sulfate groups were replaced by *O*-sulfate groups, hydroxyls were methylated in the A domain, and the T domain was built up with repeating units of 2,6-di-*O*-sulfonato β-d-glucose. The thrombin assays were performed with regioisomers of comparable chain length. When the T domain is at the reducing end, it was established that the thrombin inhibition is about 30 times less potent than when it is located at the non-reducing end (IC_50_ = 164 vs. 5 ng/mL, comparison made respectively with an octadecasaccharide and a heptadecasaccharide). 

While heparin does contain sequences such as described in Figure 7, the overall picture of polysaccharide structures able to inhibit thrombin is more diverse, and the richness of activity of this collection of biosynthetic macromolecules is certainly not uniquely explained by such structures. As a matter of fact, the chromatographic complexity of the octadecasaccharide fractions speak to the diversity (Figure 2), just as well as the unexpected series of double AT binding octadecasaccharides regioisomers studied herein (Figure 1). For these, the aFXa and aFIIa were assayed by chromogenic assay and are reported in the Table 5.

Regarding aFXa inhibitory potency, these series are on molar basis 1.5 to 2.6 times more potent than the fondaparinux reference (1480 UI/µmol). This is in agreement with previous studies performed on dodecasaccharides and tetradecasaccharides where the same phenomenon was observed [32]. As a consequence, this further emphasizes that the double-site oligosaccharides are able, through a dynamic equilibrium, to engage both AGA*IA sites with antithrombin and to increase by about twofold the molar aFXa activity. At this stage however, more subtle interpretation of their ranking is difficult to assess as it is established that several parameters, either synergistic or antagonistic, might influence their potency. Among them, we could highlight the spacer length between two AGA*IA sequences, the nature of AGA*IA flanking oligosaccharides as well as their location either at RE or NRE. 

Regarding thrombin activity, when the T-Domain flanking units are located at the reducing end, the potency is between 5 and 9 IU/mg. This is very low when compared to the NLT 180 IU/mg of USP Heparin. However, when T-domain flanking units are at the NRE, the thrombin activity is increasingly noteworthy. Interestingly, the octadecasaccharides **4** and **5** have the same T domain (ΔIs-IdoA) followed by an AGA*IA at the NRE but the aFIIa activities are respectively 22.6 and 81.5 IU/mg. In this case, the location of the reducing end AGA*IA plays an important role. For compound **4**, the RE pentasaccharide is not completely at the end of the chain, whereas for **5**, it is fully located at the reducing end of the octadecasaccharide. This sequence configuration provides the opportunity to maximize the chain length between at least one AGA*IA and the T-domain, which in turn strengthens the ternary complex with antithrombin and thrombin. 

The octadecasaccharide **6** possesses three consecutive Is disaccharides at the NRE and one AGA*IA at the RE. This configuration maximizes both the anionic charge density for the T-domain and the spacer chain length with the reducing end AGA*IA pentasaccharide. As a consequence, the thrombin inhibition is the highest one observed in these series, with a value of 119.3 IU/mg. We conclude that between the regioisomers **2** and **6**, the thrombin inhibition potency increases by about 20 times, which is in good alignment with the results obtained on synthetic compounds. However, there are still some open questions remaining, especially when octadecasaccharide **1** is compared with octadecasaccharides **5** and **6**. For these three octadecasaccharides, there is one AGA*IA sequence fully located at the reducing end. Their differences lie in the T domain. The last two disaccharides could be considered to interact with thrombin and stabilize the complex according to Petitou et al. [19]. For octadecasaccharides **1**, **5** and **6**, there is respectively four, four and six sulfate groups. The charge density influence on thrombin inhibition could be expected for octadecasaccharides **5** and **6** but does not correlate for octadecasaccharide **1**, which is at least 10 times less potent than **5** and **6**. If we take into account that the T-domain is also an AGA*IA sequence, antithrombin could compete with thrombin and disfavor the ternary complex formation, which in turn might explain the very low potency of octadecasaccharide **1**. 

## 3. Materials and Methods

### 3.1. Materials and Chemicals

Semuloparin was obtained from Sanofi (Vitry sur Seine, France). This product was prepared according to Example 2 described in a U.S. patent [33]. All other reagents and chemicals were of the highest quality available. Water was purified with a Millipore Milli-Q purification system. *Flavobacterium heparinum* heparinase I (EC 4.2.2.7) and Δ^4,5^-Glucuronate-2-sulfatase were obtained from Grampian Enzymes (Aberdeen, UK). AT used for the determination of biological activities was freeze-dried material of human origin (Instrumentation Laboratory, Le Pre Saint Gervais, France). FXa (71-nKat flask) was freeze-dried material of bovine origin (Instrument Laboratory). FIIa was freeze-dried material of human origin (Stago, Asnières sur Seine, France). FXa (S2765, Instrumentation Laboratory) and FIIa (S2238, Instrumentation Laboratory) substrates were freeze-dried materials.

### 3.2. Chromatographic Methods

Following the method in reference [12,17], 150 mm × 2.1 mm columns filled with Kinetex C_18_ 2.6 µm particles (Phenomenex, Le Pecq, France) were used after the CTA dynamic coating. A linear concentration gradient (0–2 M) of aqueous ammonium methane sulfonate, pH 2.5 was applied. 

For the analytical control of collected fractions or chromatographic separations, a Carbopack AS11 column (250 mm × 2.1 mm) (Thermo Scientific Dionex, Courtaboeuf, France) was used with aqueous NaClO_4_ mobile phase. 

LC/MS with an ion-pairing chromatographic system (Acquity UPLC BEH C_18_ column, 2.1 mm × 150 mm, 1.7 μm, Waters, Saint-Quentin-en-Yvelines, France) was the third orthogonal method applied for deciphering the complex mixture [14,17]. The method initially described in [34] and adapted in [14] was broadened [17] to determine the number of ion-pair adducts included in the molecular ions. Briefly, each separation was duplicated, one using pentylamine (PTA) as ion pairing reagent and the second one using hexylamine (HXA). The number of adducts could be deduced from the mass shift in ion peaks obtained in parallel with both conditions. Partial degradation of 3-O sulfated glucosamines located at the reducing end was detected when working at 40 °C, and therefore, the operating column temperature was lowered to 30 °C for sequencing experiments. 

### 3.3. Procedure for Isolation of Octadecasaccharides

The octadecasaccharide fraction was purified by gel permeation chromatography (GPC) from semuloparin as previously described [17]. 1.85 g of semuloparin were directly injected on a system equipped with two columns (100 × 5 cm (I.D.) connected in series, packed with polyacrylamide gel (Bio Gel P-30, Fine, Bio Rad, Marnes-la-Coquette, France)), and circulated at 1.7 mL/min with NaClO_4_ 0.2 M. UV detection was used at 232 nm. 600 g of semuloparin were injected in fractions over about 350 runs. Selected fractions were first concentrated, desalted on Sephadex G10 columns (100 × 7 cm) and then lyophilized. Finally, 10 g of octadecasaccharides were obtained.

#### 3.3.1. Fractionation on AT Affinity Chromatography

The octadecasaccharide fractionated by chromatography on an AT Sepharose column (30 cm × 7 cm) as previously described [17]. The column was prepared by coupling human AT (2 g) as described by Hoök et al. [35]. Octadecasaccharides (80 to 150 mg) were injected in each run. The low-affinity fraction was eluted from the column with a 0.25 M NaCl solution buffered at pH 7.4 with 1 mM Tris/HCl at 12 mL/min. The five high-affinity octasaccharide fractions were eluted with a five step gradient of NaCl (0.74 M, 1.23 M, 1.71 M, 2.2 M and 3.5 M NaCl and 1 mM Tris-HCl, pH 7.4). All fractions were desalted on Sephadex–G10. After 100 injections on AT-affinity chromatography, 4.2 g were collected for the low-affinity fraction, 800 mg for F1, 1.57 g for F2, 1.02 g for F3, 616 mg for F4 and 385 mg for F5.

#### 3.3.2. Purification of Octadecasaccharide Fraction F3

Focus was given to the separation of octadecasaccharides regioisomers with 2 AGA*IA sequences positioned differently within the chain (Figure 1). These are the components of major interest in the affine fraction F3 (Figure 2). The five octadecasaccharide series were purified by combining three chromatographic methods with orthogonal selectivities following the previously described procedure [17] namely, CTA-SAX, AS11 and ion pairing chromatography. We distinguish herein two distinct types of octadecasaccharides to guide the separation strategy. Type I octadecasaccharides (**2** and **3**) are devoid of 2-O sulfation at the non-reducing end’s uronic acid whereas Type II compounds (**4**, **5** and **6**) are 2-O sulfated (Figure 1). The 2-O sulfation influences the maximum of the UV spectrum and the retention behavior in CTA-SAX and AS11 chromatography. The details of the procedure are reported in the Supplementary Data. 

### 3.4. Structural Characterization by Enzymatic Sequencing

The sequencing of octadecasaccharides (10 to 20 µg) was done by controlled depolymerisation with heparinase 1 from *Flavobacterium heparinum* following the described procedure [12,17]. The first step for Type I oligosaccharides was reduction by NaBH_4_, used to selectively label the reducing end disaccharide [17]. For Type II octadecasaccharides, Δ^4,5^-glucuronate-2-sulfatase was initially applied to differentiate the non-reducing end, before the NaBH_4_ reduction. The reaction was initiated by the addition of 10 µL of a 0.5 UI/mL solution of sulfatase diluted in KH_2_PO_4_ 10 mM pH 7 and added in combination with 2 mg/mL of bovine serum albumin (BSA). The advancement of the 2-O desulfatation was followed by analytical CTA-SAX chromatography. At the end of the reaction, the residual enzyme was destroyed by a 2 min boiling step before performing NaBH_4_ reduction. After vacuum evaporation, reduced octadecasaccharide was diluted in a 1.7 mL HPLC vial and the pH was adjusted to 7 by addition of diluted ammonia or hydrochloric acid. Heparinase I was added (2 µL of a 0.5 UI/mL solution) and depolymerisation was performed at 16 °C. The monitoring of the fragmentation was done by injection on CTA-SAX and AS11 analytical columns. Injection in LC/MS was also performed to confirm the structure of fragments.

### 3.5. Mass Spectrometry Conditions

ESI mass spectra were obtained using a Waters Xevo Q-Tof mass spectrometer. The electrospray interface was set in positive ion mode with a capillary voltage of 2000 V and a sampling cone voltage of 20 V. The source and the desolvation temperatures were respectively 120 and 400 °C. Nitrogen was used as desolvation (750 L/min) and cone gas (25 L/min). The mass range used was 200–2000 Da (scan rate 0.5 s).

### 3.6. NMR Spectroscopy

The octadecasaccharide sample (1 mg) was dissolved in D_2_O, freeze-dried with a final dissolution in D_2_O (99.96% Euriso-Top, Saint-Aubin, France) and transferred into a 3-mm nuclear magnetic resonance (NMR) sample tube. One-dimensional and two-dimensional (2D) NMR (^1^H-^1^H and ^1^H-^13^C) spectra were recorded on a 600 MHz AVANCE II spectrometer (Bruker, Wissembourg, France) operating at 599.80 MHz using a 5-mm TXI cryoprobe with standard pulse sequences and a probe temperature of 30 °C. Proton spectra were recorded in 256 scans with a 2 s presaturation and a full recycle time of 6.5 s. The resolution prior zero filling was 0.2 Hz/pt. 2D-COSY and 2D-TOCSY with a mixing time value of 120 ms were acquired using 64 scans per series of 4 K × 512 W with a resolution of 1.16 Hz/pt before zero filling (4 K × 2 K) and appropriate apodization was applied prior to Fourier transformation. The 2D-nuclear Overhauser enhancement spectroscopy (NOESY) was performed in similar way with a mixing time value of 300 ms. The 2D-HSQC was performed using 64 scans per series of 1.5 K × 512 W with a resolution of 3.28 Hz/pt before zero filling (2 K × 1 K). Spectrum calibration was achieved by setting as external standard reference trimethylsilyl 2,2′3,3′-tetradeuteropropionoic acid (TSP-D_4_) signal to 0 ppm.

### 3.7. Anti-Factor IIa Activity and Anti-Factor Xa Activity

AT solution of 1 IU/mL were prepared in pH 7.4 PEG 6000 buffer (0.1% PEG 6000 in pH 7.4 buffer [50 mM Tris and 150 mM NaCl adjusted by HCl to pH 7.4]). FXa was prepared to 1.8 nkat/mL in pH 7.4 PEG6000 buffer. FIIa was prepared to 5 IU/mL in pH 7.4 PEG6000 buffer. The chromogenic substrate solution for FXa and FIIa was prepared to a final concentration of 0.5 mM in pH 8.4 buffer (50 mM Tris, 175 mM NaCl, and 7.5 mM edetate disodium adjusted by HCl to pH 8.4). Semuloparin biological standard (3000ET with assigned potencies of 101.4 IU/mL for anti-FXa and 1.8 IU/mL for anti-FIIa), calibrated against the second LMWH international standard (NIBSC, code 01/608), was used for calibration.

For anti-FXa titration, the semuloparin standard solution was diluted with pH 7.4 buffer to obtain target concentrations of 0.04, 0.05, 0.06, and 0.07 IU/mL. Each concentration was assessed in duplicate (two wells filled with the same diluted solution), and two independent experiments (independent dispensing and dilutions) were conducted.

For anti-FIIa titration, the semuloparin standard solution was diluted with pH 7.4 buffer to obtain target concentrations of 0.007, 0.008, 0.010, and 0.012 IU/mL. Each concentration was assessed in duplicate (two wells filled with the same diluted solution), and three independent experiments (independent dispensing and dilutions) were conducted.

Octadecasaccharide solution was prepared to a final concentration with activities in IU/mL close to the semuloparin standard. The solution was then diluted and dispensed as for the standards.

Plates were heated at 37 °C. Solutions for analysis (20 µL) were pipetted into a 96-well plate, and 20 µL of AT solution was added. Samples were then mixed and left for 60 s before 40 µL of the FXa (or FIIa) solution were added, and the solutions were mixed and left for a further 60 s. Next, 100 µL of chromogenic substrate solution were added, mixed, and left for 240 s. Finally, 100 µL of 42% acetic acid solution were added, mixed, and left for 60 s before the optical density at 405 nm was read.

The internally developed software “Parallel”, running on the Statistical Analysis System (SAS version 9.1, SAS France, Grégy-sur-Yerres, France)), was used for statistical analysis according to the European Pharmacopoeia Ph. Eur. § 5.3 for parallel-line models. Each assay result was the mean average of the independent valid tests.

## 4. Conclusions

The results of this study, while confirming the structure-activity relationship on synthetic compounds, also demonstrate that the overall picture for thrombin inhibition compounds may be much more complex in natural heparin sequences as well as for LMWH products. We have demonstrated that for octadecasaccharide regioisomers, built up from the same AGA*IA sequences and Is flanking disaccharides, thrombin inhibition potency may dramatically differ. Indeed, when the AGA*IA sequences are located at the reducing end vs. the non-reducing end, the octadecasaccharide becomes 20 times more potent in aFIIa activity (compound **6** vs. **2**). This is in agreement with the previous findings reported on synthetic compounds by Petitou et al. [19,20,21]. However, even if an AGA*IA sequence is borne at the reducing end, the presence of another one at the non-reducing end (compound **1**) obviously destabilize the complex with thrombin and decrease the potency by one log of magnitude order. The anionic charge density of T-domain is an important factor for thrombin binding but its ability to interact strongly with other proteins should be taken into consideration to improve our prediction of structure activity relationships. The findings developed in this paper pointed out that for octadecasaccharides regioisomers the sequence arrangement is critical for interaction with proteins regulating the coagulation cascade. This is emphasizing the role and the potential impact of the selectivity of the heparin depolymerization process on the overall biological properties of LMWH.

## Figures and Tables

**Table 1:** Structural symbols.

	Structural Symbols	
ΔIVa = ΔU-GlcNAc	-IVa_id_- = -IdoA-GlcNAc-	-IVa_glu_- = -GlcA-GlcNAc-
ΔIVs = ΔU-GlcNS		-IVs_glu_- = -GlcA-GlcNS-
ΔIVs = ΔU-GlcNS,3S		-IVs_glu_- = -GlcA-GlcNS,3S-
ΔIIa = ΔU-GlcNAc,6S	-IIa_id_- = -IdoA-GlcNAc,6S-	
ΔIIIa = ΔU2S-GlcNAc	-IIIa_id_- = -IdoA2S-GlcNAc-	
ΔIIs = ΔU-GlcNS,6S		-IIs_glu_- = -GlcA-GlcNS,6S-
ΔIIs = ΔU-GlcNS,3S,6S		-IIs_glu_-= -GcA-GlcNS,3S,6S-
ΔIIIs = ΔU2S-GlcNS	-IIIs_id_- = -IdoA2S-GlcNS-	
ΔIa = ΔU2S-GlcNAc,6S	-Ia_id_- = -IdoA2S-GlcNAc,6S-	
ΔIs = ΔU2S-GlcNS,6S	-Is_id_- = -IdoA2S-GlcNS,6S-	
ΔIs = ΔU2S-GlcNS,3S,6S	-Is_id_- = -IdoA2S-GlcNS,3S,6S-	

**Table 2:** Heparinase 1 cleaving sites (**^↓^** major sites; ^↓^ minor sites).

	Structure	Heparinase I Cleaving Sites
Octadeca. 2	ΔIIa-IIs_glu_-Is_id_-IIa_id_-IIs_glu_-Is_id_-Is_id_-Is_id_-Is_id_	ΔIIa-IIs_glu_**^↓^**Is_id_-IIa_id_-IIs_glu_**^↓^**Is_id_^↓^Is_id_**^↓^**Is_id_-Is_id_^red^
Octadeca. 3	ΔIIa-IIs_glu_-Is_id_-Is_id_-IIa_id_-IIs_glu_-Is_id_-Is_id_-Is_id_	ΔIIa-IIs_glu_**^↓^**Is_id_^↓^Is_id_-IIa_id_-IIs_glu_**^↓^**Is_id_**^↓^**Is_id_-Is_id_^red^
	ΔIIa-IIs_glu_-Is_id_-Is_id_-Is_id_-IIa_id_-IIs_glu_-Is_id_-Is_id_	ΔIIa-IIs_glu_**^↓^**Is_id_^↓^Is_id_^↓^Is_id_-IIa_id_-IIs_glu_**^↓^**Is_id_-Is_id_^red^
	ΔIIa-IIs_glu_-Is_id_-Is_id_-Is_id_-Is_id_-IIa_id_-IIs_glu_-Is_id_	ΔIIa-IIs_glu_**^↓^**Is_id_^↓^Is_id_^↓^Is_id_**^↓^**Is_id_-IIa_id_-IIs_glu_-Is_id_^red^
	ΔIs-IIa_id_-IIs_glu_-Is_id_-IIa_id_-IIs_glu_-Is_id_-Is_id_-Is_id_	ΔIIs-IIa_id_-IIs_glu_**^↓^**Is_id_-IIa_id_-IIs_glu_**^↓^**Is_id_**^↓^**Is_id_-Is_id_^red^
Octadeca. 4	ΔIs-IIa_id_-IIs_glu_-Is_id_-Is_id_-IIa_id_-IIs_glu_-Is_id_-Is_id_	ΔIIs-IIa_id_-IIs_glu_**^↓^**Is_id_^↓^Is_id_-IIa_id_-IIs_glu_**^↓^**Is_id_-Is_id_^red^
Octadeca. 5	ΔIs-IIa_id_-IIs_glu_-Is_id_-Is_id_-Is_id_-IIa_id_-IIs_glu_-Is_id_	ΔIIs-IIa_id_-IIs_glu_**^↓^**Is_id_^↓^Is_id_**^↓^**Is_id_-IIa_id_-IIs_glu_-Is_id_^red^
	ΔIs-Is_id_-IIa_id_-IIs_glu_-Is_id_-IIa_id_-IIs_glu_-Is_id_-Is_id_	ΔIIs^↓^Is_id_-IIa_id_-IIs_glu_**^↓^**Is_id_-IIa_id_-IIs_glu_**^↓^**Is_id_-Is_id_^red^
	ΔIs-Is_id_-IIa_id_-IIs_glu_-Is_id_-Is_id_-IIa_id_-IIs_glu_-Is_id_	ΔIIs^↓^Is_id_-IIa_id_-IIs_glu_**^↓^**Is_id_**^↓^**Is_id_-IIa_id_-IIs_glu_-Is_id_^red^
Octadeca. 6	ΔIs-Is_id_-Is_id_-IIa_id_-IIs_glu_-Is_id_-IIa_id_-IIs_glu_-Is_id_	ΔIIs^↓^Is_id_**^↓^**Is_id_-IIa_id_-IIs_glu_**^↓^**Is_id_-IIa_id_-IIs_glu_-Is_id_^red^

**Table 3:** Key fragments for octadecasaccharides.

	**Key Fragments for Type I Octadecasaccharides**
Octadecasaccharide **2**	ΔIIa-IIs_glu_-Is_id_-IIa_id_-IIs_glu_; ΔIs-Is_id_^red^
Octadecasaccharide **3**	ΔIIa-IIs_glu_-Is_id_-Is_id_-IIa_id_-IIs_glu_; ΔIs-Is_id_^red^
	**Key Fragments for Type II Octadecasaccharides**
Octadecasaccharide **4**	ΔIIs-IIa_id_-IIs_glu_; ΔIIs-IIa_id_-IIs_glu_-Is_id_-Is_id_-IIa_id_-IIs_glu_; ΔIs-Is_id_^red^
Octadecasaccharide **5**	ΔIIs-IIa_id_-IIs_glu_; ΔIs-IIa_id_-IIs_glu_-Is_id_^red^
Octadecasaccharide **6**	ΔIIs; ΔIIs-Is_id_; ΔIIs-Is_id_-Is_id_-IIa_id_-IIs_glu_; ΔIs-IIa_id_-IIs_glu_-Is_id_^red^

**Table 4:** Proton and carbon chemical shifts of the two pentasaccharide sequences identified inside octadecasaccharide **6**.

	**ΔIs**		**Is_id_**		**Is_id_**	
	**ΔU_2S_**	**A_NS,6S_**	**I_2S_**	**A_NS,6S_**	**I_2S_**	**A_NS,6S_**
1	5.51/98.4	5.40/98.1	5.21/100.4	5.41/97.8	5.22/100.4	5.34/96.6
2	4.62/75.7	3.30/58.9	4.34/76.9	3.27/59.0	4.35/76.9	3.26/59.0
3	4.31/64.0	3.65/70.8	4.22/70.2	3.66/70.8	4.20/70.4	3.65/70.8
4	5.98/107.1	3.84/79.3	4.10/77.5	3.76/77.1	4.11/77.1	3.78/77.2
5		4.03/70.0	4.81/70.5	4.02/70.3	4.81/70.5	3.96/70.4
6,6′		4.36/4.26 67.4		4.40/4.27 67.5		4.41/4.26 67.5
CH_3_						
Pentasaccharide 1						
	**IIa_id_**		**IIs_glu_**		**Is_id_**	
	**I**	**A_Nac,6S_**	**G**	**A_NS,3S,6S_**	**I_2S_**	**A_NS,6S_**
1	5.01/103.1	5.39/98.1	4.60/102.2	5.51/97.2	5.18/100.6	5.34/96.6
2	3.78/69.8	3.93/55.0	3.38/74.6	3.45/57.8	4.32/78.1	3.26/59.0
3	4.12/68.9	3.77/70.7	3.70/77.5	4.37/77.4	4.17/71.6	3.66/70.8
4	4.08/75.8	3.73/78.5	3.80/77.8	3.97/74.1	4.14/77.1	3.79/77.7
5	4.79/69.8	4.02/70.4	3.76/78.3	4.16/70.7	4.79/71.2	3.96/70.4
6,6′		4.34/4.21 67.4		4.49/4.26 67.1		4.46/4.23 67.4
CH_3_		2.05/23.2				
Pentasaccharide 2						
	**IIa_id_**		**IIs_glu_**		**Is_id_**	
	**I**	**A_Nac,6S_**	**G**	**A_NS,3S,6S_**	**I_2S_**	**A_NS,6S_**
1	5.01/103.1	5.39/98.1	4.60/102.2	5.53/97.1	5.18/100.6	4.45/92.2
2	3.78/69.8	3.93/55.0	3.39/74.6	3.45/57.8	4.31/78.1	3.27/59.0
3	4.12/68.9	3.77/70.7	3.70/77.5	4.36/77.4	4.17/71.6	3.70/70.6
4	4.08/75.8	3.73/78.5	3.80/77.8	3.97/74.1	4.16/77.2	3.78/77.6
5	4.79/69.8	4.02/70.4	3.76/78.3	4.17/70.7	4.74/71.5	4.13/69.7
6,6′		4.34/4.21 67.4		4.49/4.26 67.1		4.43/4.30 68.0
CH_3_		2.05/23.2				

Note: Listed as H/C in ppm.

**Table 5:** aFXa and aFIIa activities of isolated octadecasaccharides.

	Structure	aFXa Activity (IU/mg)	aFXa Activity (IU/µmol)	aFIIa Activity (IU/mg)
Octadecasaccharide **1**	ΔIIa-IIs_glu_-Is_id_-IIa_id_-IIs_glu_-Is_id_-IIa_id_-IIs_glu_-Is_id_	562	3090	8.8
Octadecasaccharide **2**	ΔIIa-IIs_glu_-Is_id_-IIa_id_-IIs_glu_-Is_id_-Is_id_-Is_id_-Is_id_	371	2100	5.9
Octadecasaccharide **3**	ΔIIa-IIs_glu_-Is_id_-Is_id_-IIa_id_-IIs_glu_-Is_id_-Is_id_-Is_id_	442	2502	9
Octadecasaccharide **4**	ΔIs-IIa_id_-IIs_glu_-Is_id_-Is_id_-IIa_id_-IIs_glu_-Is_id_-Is_id_	540	3057	22.6
Octadecasaccharide **5**	ΔIs-IIa_id_-IIs_glu_-Is_id_-Is_id_-Is_id_-IIa_id_-IIs_glu_-Is_id_	517	2927	81.5
Octadecasaccharide **6**	ΔIs-Is_id_-Is_id_-IIa_id_-IIs_glu_-Is_id_-IIa_id_-IIs_glu_-Is_id_	705	3991	119.3

## Supplementary Materials

Supplementary materials are available online.

## Abbreviations

AT, antithrombin; FXa, factor Xa; FIIa, factor IIa or thrombin; SAX, strong anion exchange; CTA-SAX, cetyltrimethylammonium strong anion exchange; LMWH, low-molecular-weight heparin; GPC, gel permeation chromatography; PTA, pentylamine; HXA, hexylamine; HFIP, 1,1,1,3,3,3-hexafluoro-2-propanol; LC/MS, liquid chromatography–mass spectrometry; DQF-COSY, double-quantum-filter correlation spectroscopy; 2D-NOESY, nuclear Overhauser enhancement spectroscopy; HSQC, heteronuclear single-quantum coherence; TOCSY, total correlated spectroscopy; Glc_Nac_; *N*-acetyl-α-d-glucosamine; Glc_NAc,6S_, *N*-acetylated, 6-*O*-sulfated GlcN; Glc_NS,6S_, *N*,6-*O*-disulfated GlcN; Glc_NS,3,6S_, *N*,3,6-*O*-trisulfated GlcN; IdoA, α-l-iduronic acid; IdoA_2S_, 2-*O*-sulfated IdoA; AGA∗IA, pentasaccharide sequence of Glc_NAc,6S_-GlcA-Glc_NS,3,6S_-IdoA_2S_-Glc_NS,6S_ ; ΔU, 4,5-unsaturated uronic acid; ΔU_2S_, 2-*O*-sulfated, 4,5-unsaturated uronic acid.
