# Supplementary material for: New Insights in Thrombin Inhibition Structure–Activity Relationships by Characterization of Octadecasaccharides from Low Molecular Weight Heparin
Source: Molecules. 2017 Mar 8;22(3):428. doi: 10.3390/molecules22030428 (PMC6155232; doi:10.3390/molecules22030428)
Supplement: Supplementary file 1 [file molecules-22-00428-s001.pdf]

Supplementary data

New Insights in Thrombin Inhibition Structure-Activity Relationships by Characterization of Octadecasaccharides from Low Molecular Weight Heparin

Pierre A. J. MOURIER, Olivier Y. GUICHARD,

Fréderic HERMAN, Philippe SIZUN and Christian VISKOV*

Sanofi, 13 Quai Jules Guesde, 94403 Vitry sur Seine, France

In memoriam: The authors would like to respectfully dedicate this article to Pr. B. Casu, a pioneer in glycosaminoglycan chemistry and analysis, who passed away on November 12th, 2016.

Purification of octadecasaccharide fraction F3

The fraction is first injected in CTA-SAX chromatography on semi preparative columns (250 mm × 21 mm) filled with Betabasic C18 5 µm particles (Thermo Fisher Scientific, Villebon sur Yvette, France) and prepared following the same procedure as described in reference [1]. 50 to 100 mg of fraction 3 were injected at each run and the entire fraction was separated within 12 runs (Figure 2), 58 fractions were collected at each run. The fractions were neutralized to pH 5 to 7 by addition of ammonia. Fractions were controlled on analytical AS11 so that all fractions obtained could be pooled properly. All gathered fractions were analyzed with the LC/MS ion-pair method to guide the strategy of purification (Figure 3). In this work, a focus will be given on the separation of octadecasaccharides regioisomers with two AGA*IA sequences positioned differently in the sequence with a molecular weight of 4957 Da (Figure 1). The latest are the components of major interest in the affine fraction F3. Figure S1 shows MS spectra of these double site octadecasaccharides following the amine (PTA or HXA) used in the ion-pair. To calculate the number of amine adduct, the ion at *m/z* 2350.2 in the PTA spectrum can be assigned to $[M+nPTA+3H]^{3+}$ and in the HXA spectrum, the ion at *m/z* 2462.4 can be assigned to $[M+nHXA+3H]^{3+}$. The number of adduct is then equal to 24, confirming the molecular weight at 4957 Da.

Two types of double site were distinguished depending on the 2-O sulfatation of the unsaturated acid. This 2-O sulfation influences the maximum of the UV spectrum (Ref. [1]) and the retention behavior in CTA-SAX and AS11 chromatography. In both systems, the non 2-O sulfated types (I) are eluted first (fractions 41 to 45) and the 2-O sulfated are eluted after (types II, fractions 47 to 52). In AS11 chromatography, all regioisomers are even eluted within only two peaks, so that all octadecasaccharides of the same type are coeluted with this method.

The gathered CTA-SAX fractions were then reinjected on the CTA-SAX column after a dilution 1/5 in water. The same gradient of sodium methane sulfonate was applied. Fractions collected were controlled by analytical AS11 (Figure S2 to S5) to select those reinjected in the next purification step.

The semi-preparative scale extension of the LC/MS ion-pair method was used and 2 columns XBridge™ C18 25 cm × 1 cm, 5µm (Waters, Guyancourt, France) were connected in series. Same mobile phases as those of LC/MS were used. Mobile phase A was water and mobile phase B was water/acetonitrile (25:75) v/v. The ion pairing reagent (PTA, 15 mM) and a buffering acid, 1,1,1,3,3,3-hexafluoro-2-propanol (HFIP) (50mM) were added to both A and B. The column temperature was fixed at 40 °C and the flow rate at 4 ml/min. UV detection at 232 nm was used. The fractions collected after the CTA-SAX reinjection were pooled by 2 or 3, passed through Mega Bondelut C_{18} cartridges (Agilent Varian, Massy, France) to eliminate traces of CTA, and diluted 1/5 in water, before injection on the ion-pairing system. In the case of the CTA-SAX fraction F42 (Figure S2), the chromatograms of the ion pair separations are shown on Figure S7. LC/MS analysis of F42 is presented in Figure S6. F42 only contains double sites, essentially at molecular weight of 4957 Da. Separations obtained in the ion pair step preparative step correspond to the LC/MS selectivity. Fractions (not shown on figures) were collected rather closely, as in the two previous steps. They were neutralized immediately to pH between 5 and 7 by addition of diluted HCl. Analytical control with AS11 was realized, allowing an easy recognition of double sites from the other octadecasaccharides which were then injected on the analytical ion pair system. Since only UV detection at 232 nm (no MS) was available at that step, the detection sensitivity was lowered by the PTA in the mobile phase so that this type of control could only differentiate pure compounds and mixtures. Similar fractions (pure compounds and mixtures) obtained were gathered, vacuum evaporated to eliminate acetonitrile and reinjected on the ion pair system. At the end of the process, purified compounds were gathered and vacuum evaporated. $NaClO_4$ was added to the aqueous residual phase with to reach a 1 M concentration and passed through a Mega Bondelut C_{18} cartridge to eliminate the ion pairing agent and HFIP. The fraction was then desalted on a Sephadex G10 column (30 cm × 5 cm), ready for the last step on the AS11 semi preparative column. This method is not very selective, but its mobile phase, $NaClO_4$, is very easily eliminated in the desalting step. Here, its main object is to complete the elimination of residual reagents such as CTA PTA, HXA and HFIP. Collected fractions were controlled on analytical AS11 and vacuum concentrated before final desalting and lyophilysation. From the CTA-SAX fraction 42, two products were purified (Figure S7). The first one (peak 1) was finally identified as the octadeca-2 on Figure 1 (ΔIIa-$\underline{\text{IIs}_{\text{glu}}}$-$\text{Is}_{\text{id}}$-$\text{IIa}_{\text{id}}$-$\underline{\text{IIs}_{\text{glu}}}$-$\text{Is}_{\text{id}}$-$\text{Is}_{\text{id}}$-$\text{Is}_{\text{id}}$-$\text{Is}_{\text{id}}$) while the second one was a mixture of 2 type (I) octadecasaccharides.

For the CTA-SAX fraction F44, the injections of the selected fractions on the ion pair semi preparative system are shown in Figure S8. The last step of the purification on the AS11 column is identical to the previously described for F42. One octadecasaccharide (indicated by an arrow in Figure S8) was purified and further identified as the octadecasaccharide **3**.

For type (II) double sites, the purification was more complex. six regioisomers were present, closely eluted in fractions F49 to 52, and less abundant than type I. Indeed, as a general rule, the selectivity of the depolymerisation in semuloparin favors the presence of AGA*IA at the beginning of the oligosaccharidic chain (ΔIIa-$\underline{\text{IIs}_{\text{glu}}}$-$\text{Is}_{\text{id}}$…). The LC/MS analysis of fraction F49 is shown in Figure S9. Five type II double sites were detected. The reinjection of F49 in CTA-SAX is shown in Figure S4. Only fractions f2 to f13 were selected for the ion pair separation (Figure S10). AS11 and ion pair analytical control were applied. The last AS11 purification step was performed and finally, two purified type II double site octadecasaccharides were obtained. The first one has been identified as the octadecasaccharide **4** and the second one as the octadecasaccharide **5**.

F50 is the last fraction treated for the purification of double sites regioisomers. LC/MS is shown in Figure S11. The same procedure is applied as for previous fractions. Fractions f2 to f10 of the CTA-SAX step were selected for the ion pair step (Figure S12). After the last AS11 chromatography, two fractions were purified. The first one remains a mixture of two type (II) octadecasaccharides while the second one was octadecasaccharide **6**.

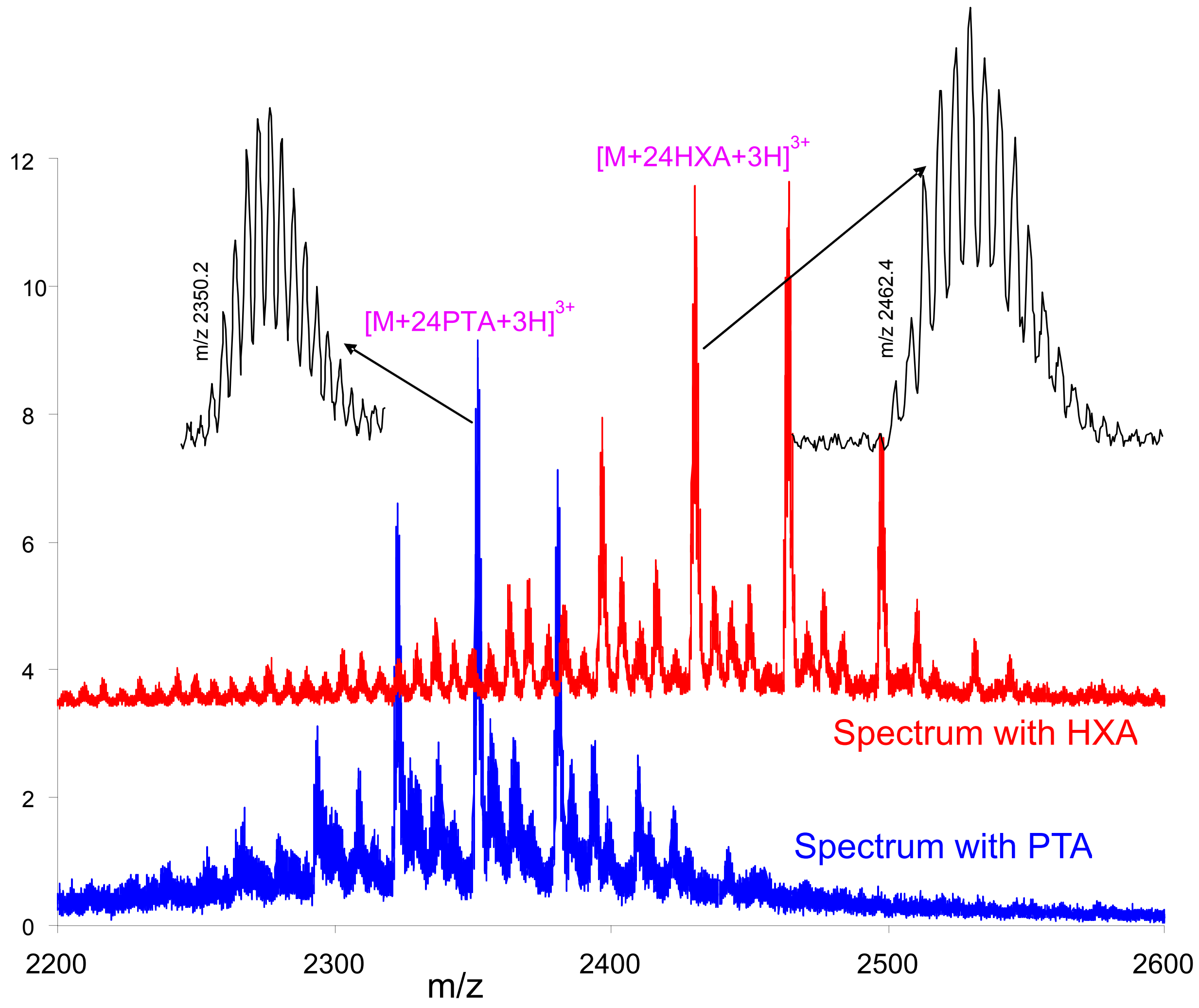

Figure S1. MS spectrum of octadecasaccharide **2**.

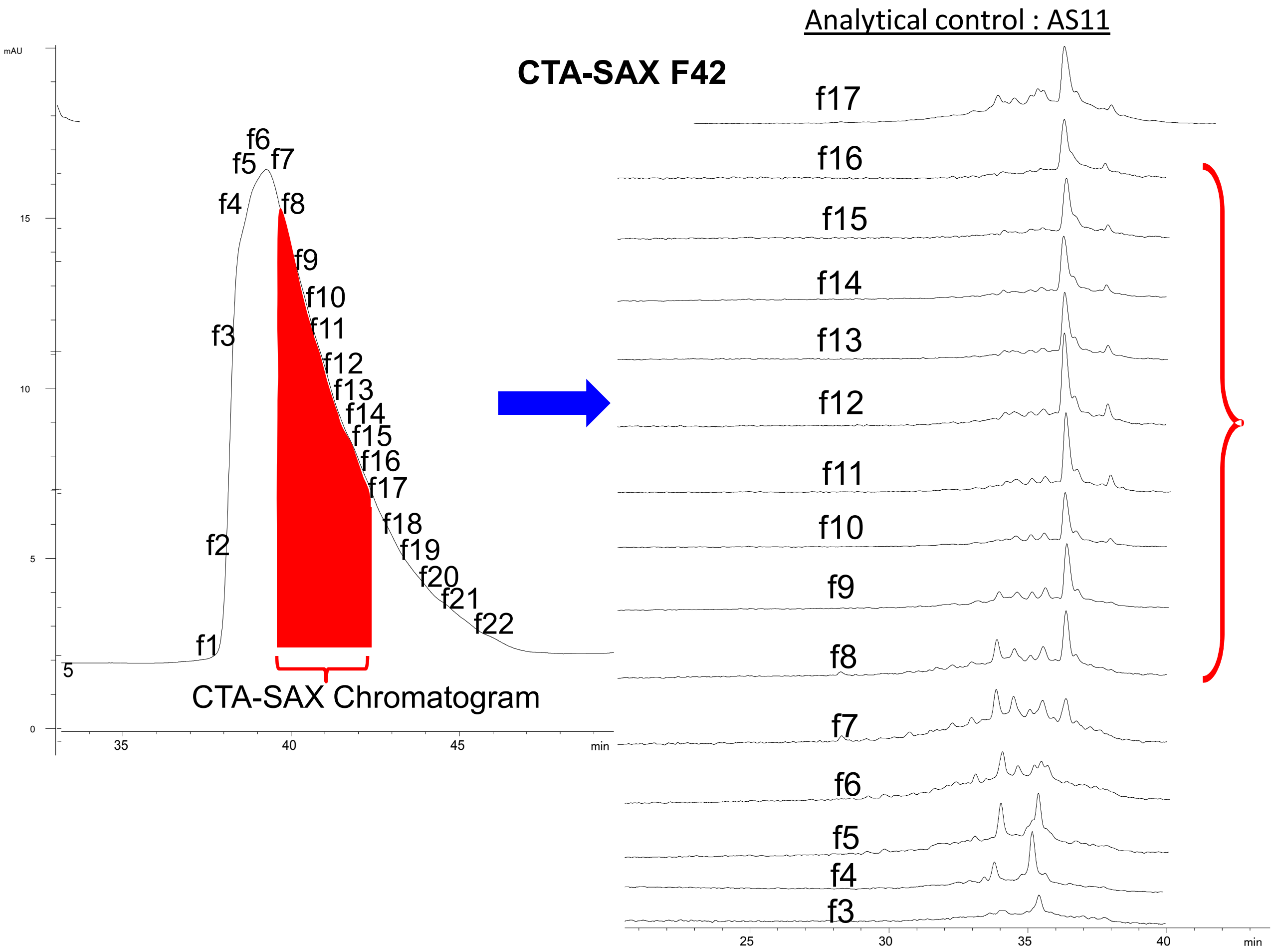

Figure S2. Reinjection of fraction F42 on the same CTA-SAX semi-preparative column and analytical control of the fractions on AS11 columns.

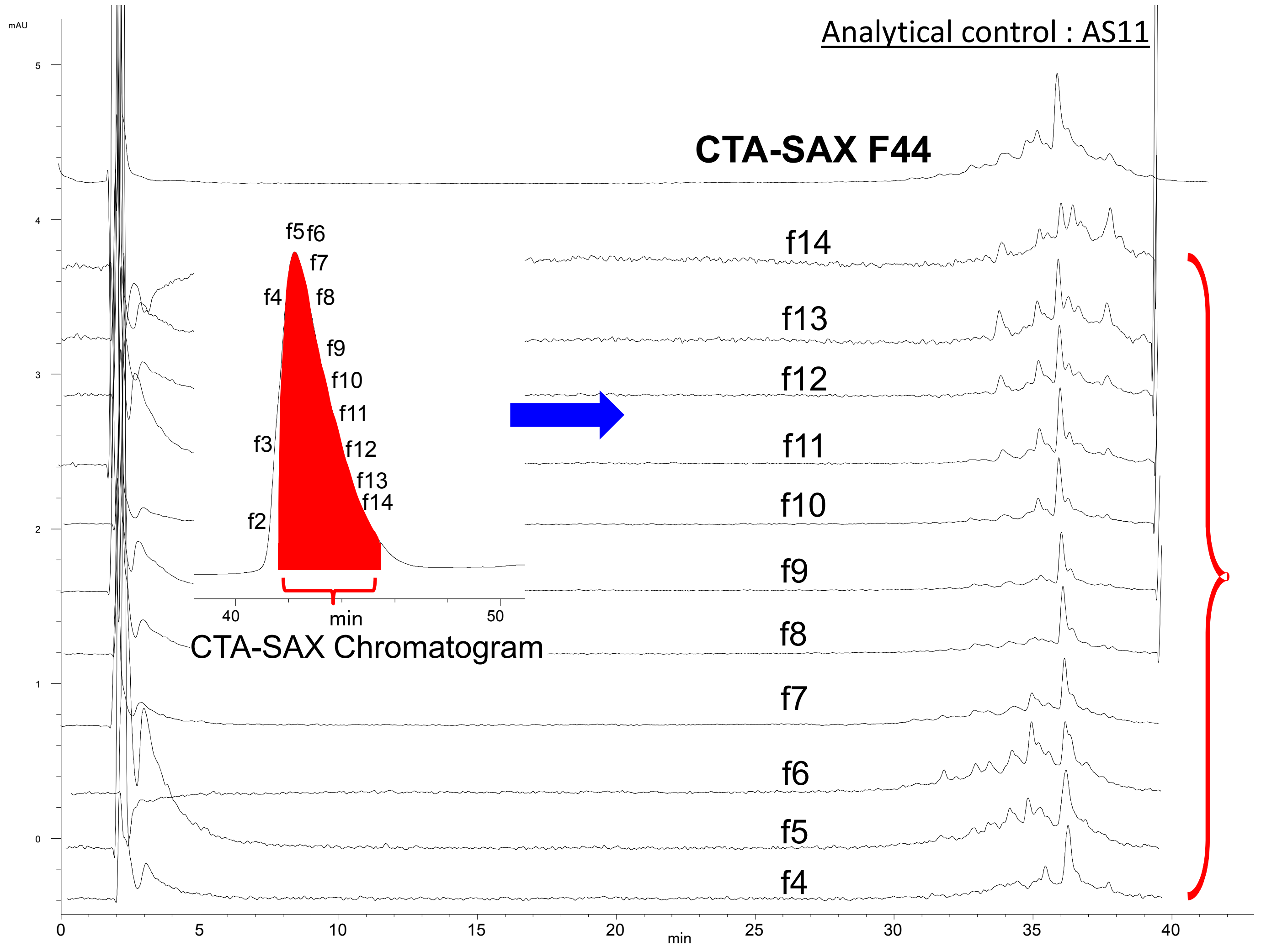

Figure S3. Reinjection of fraction F44 on the same CTA-SAX semi-preparative column and analytical control of the fractions on AS11 columns.

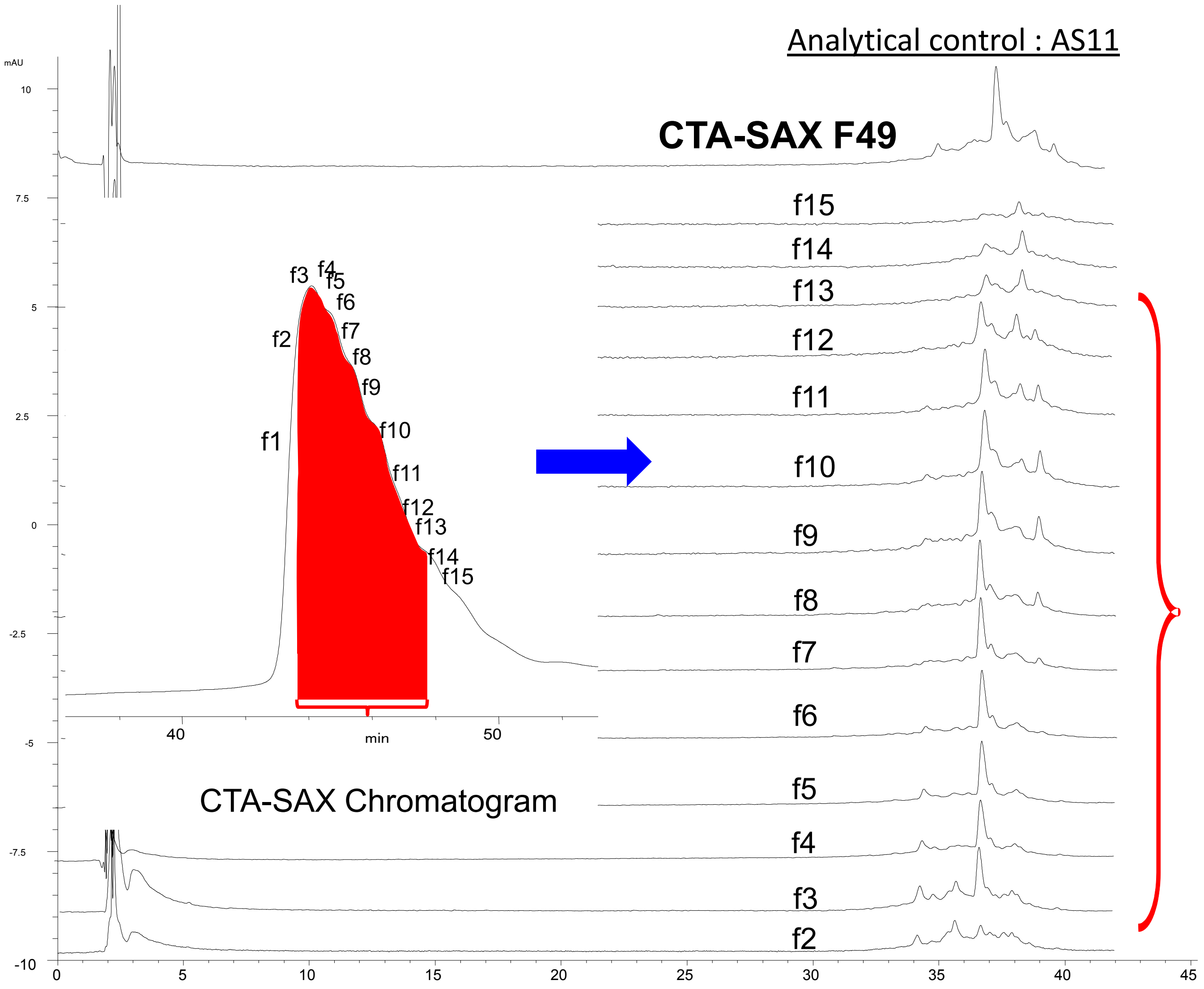

Figure S4. Reinjection of fraction F49 on the same CTA-SAX semi-preparative column and analytical control of the fractions on AS11 columns.

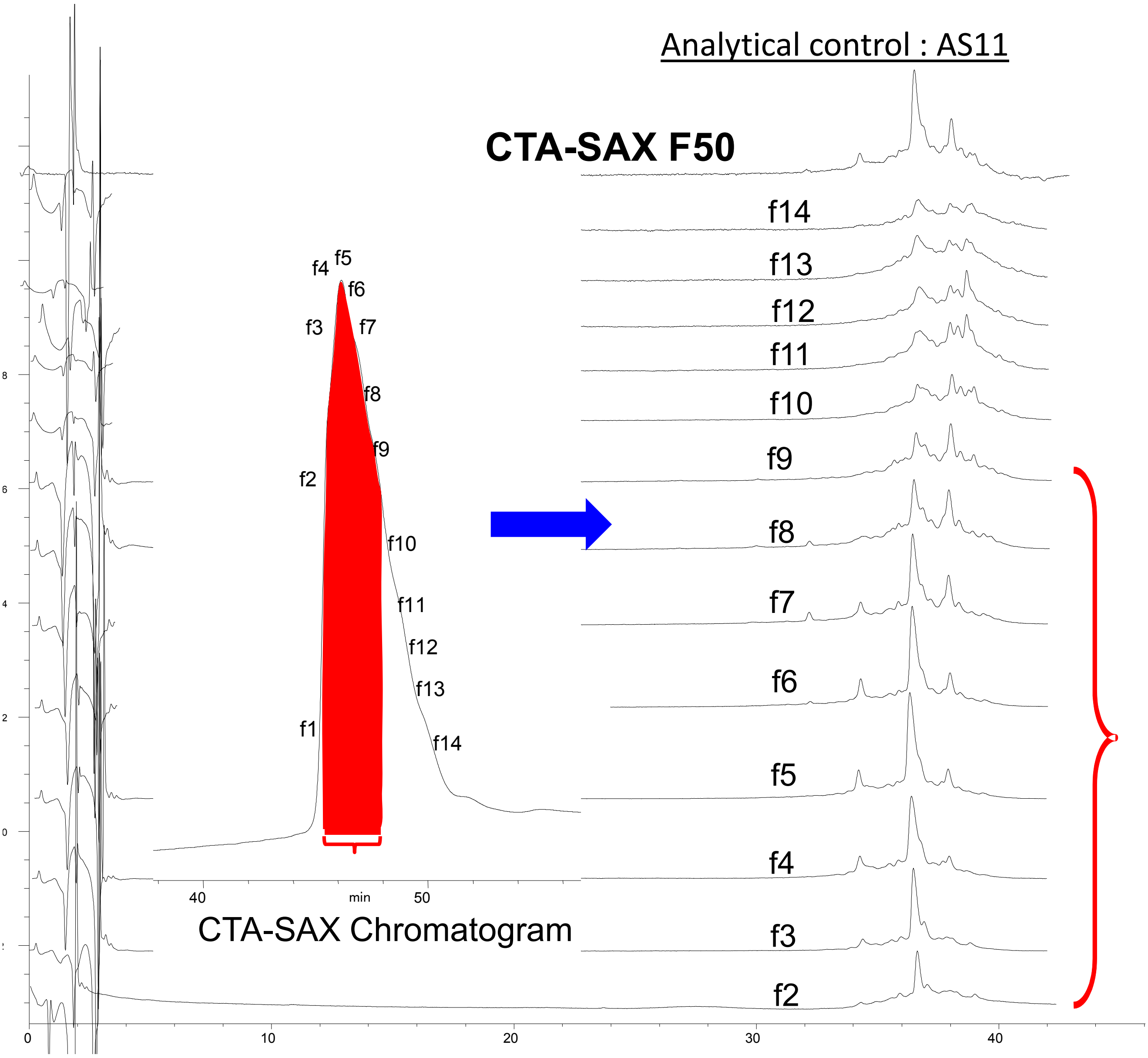

Figure S5. Reinjection of fraction F50 on the same CTA-SAX semi-preparative column and analytical control of the fractions on AS11 columns.

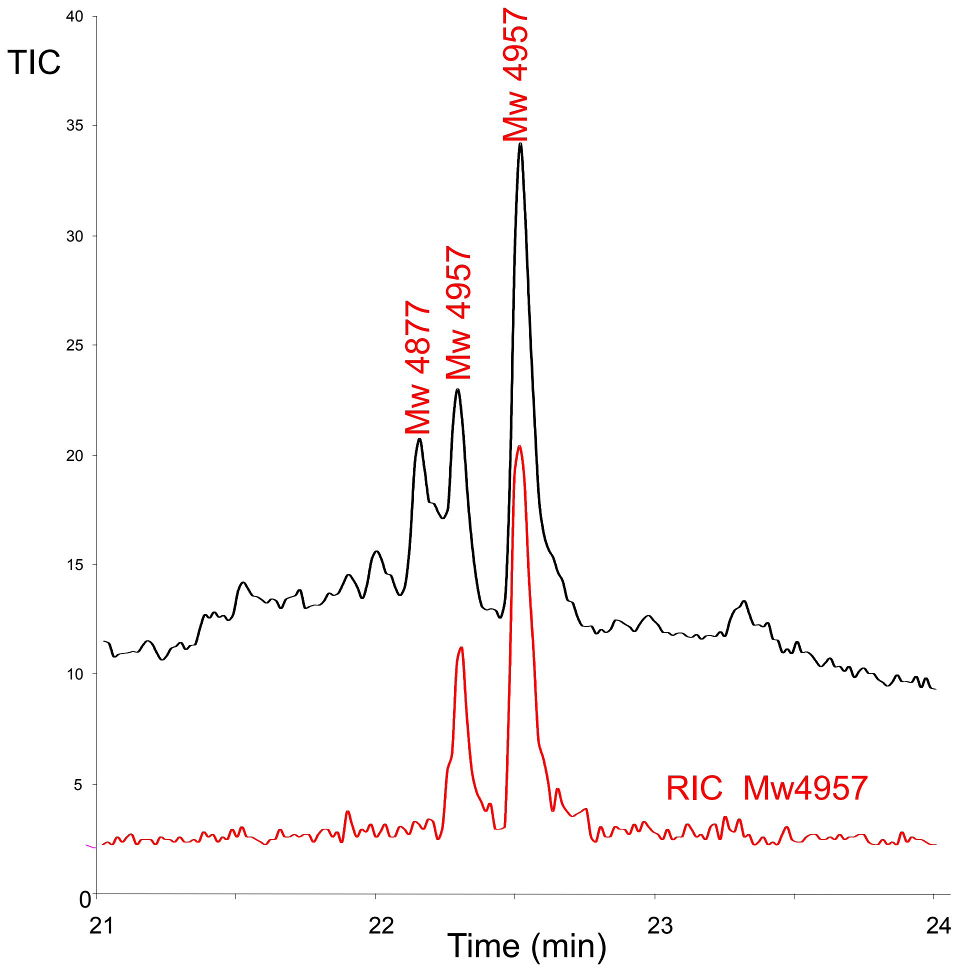

Figure S6. LC/MS of the fraction F42.

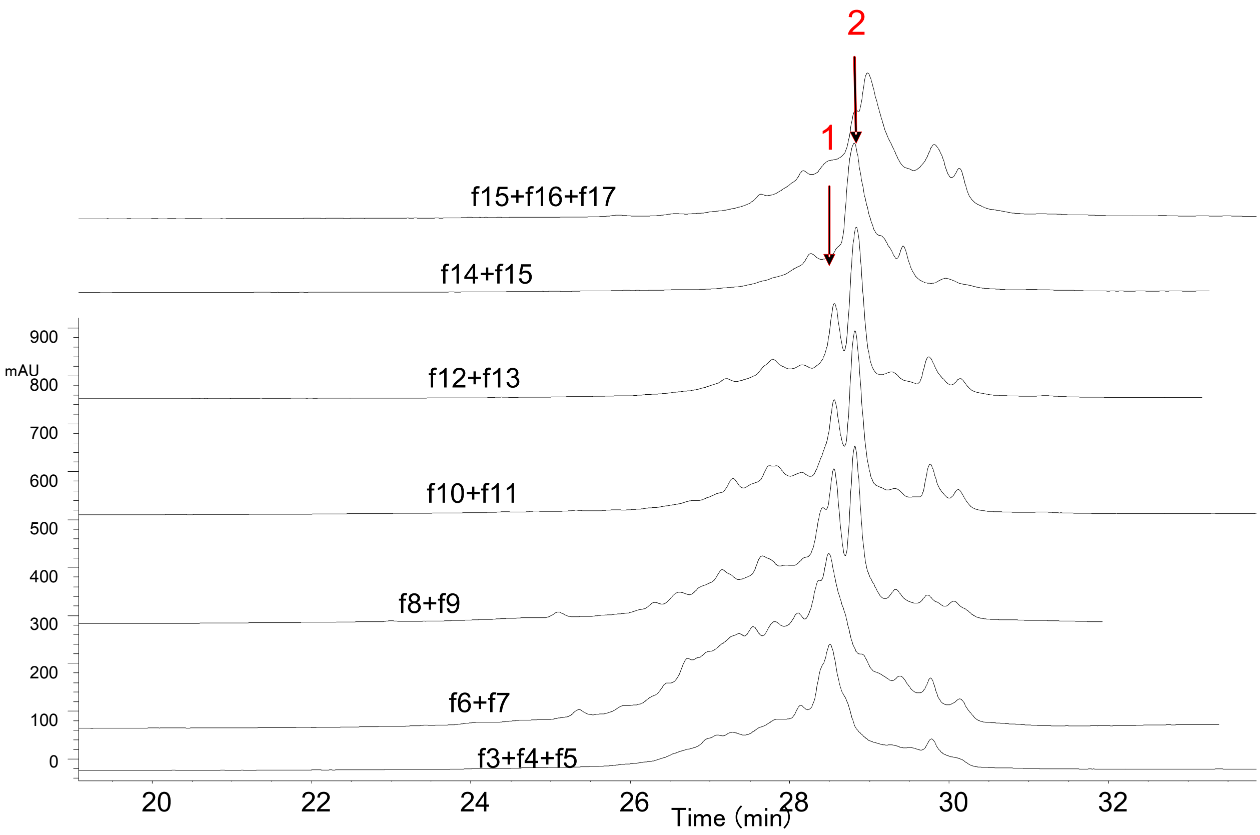

Figure S7. Injection of the fractions selected in the last step for purification of F42 in semi-preparative ion pair chromatography.

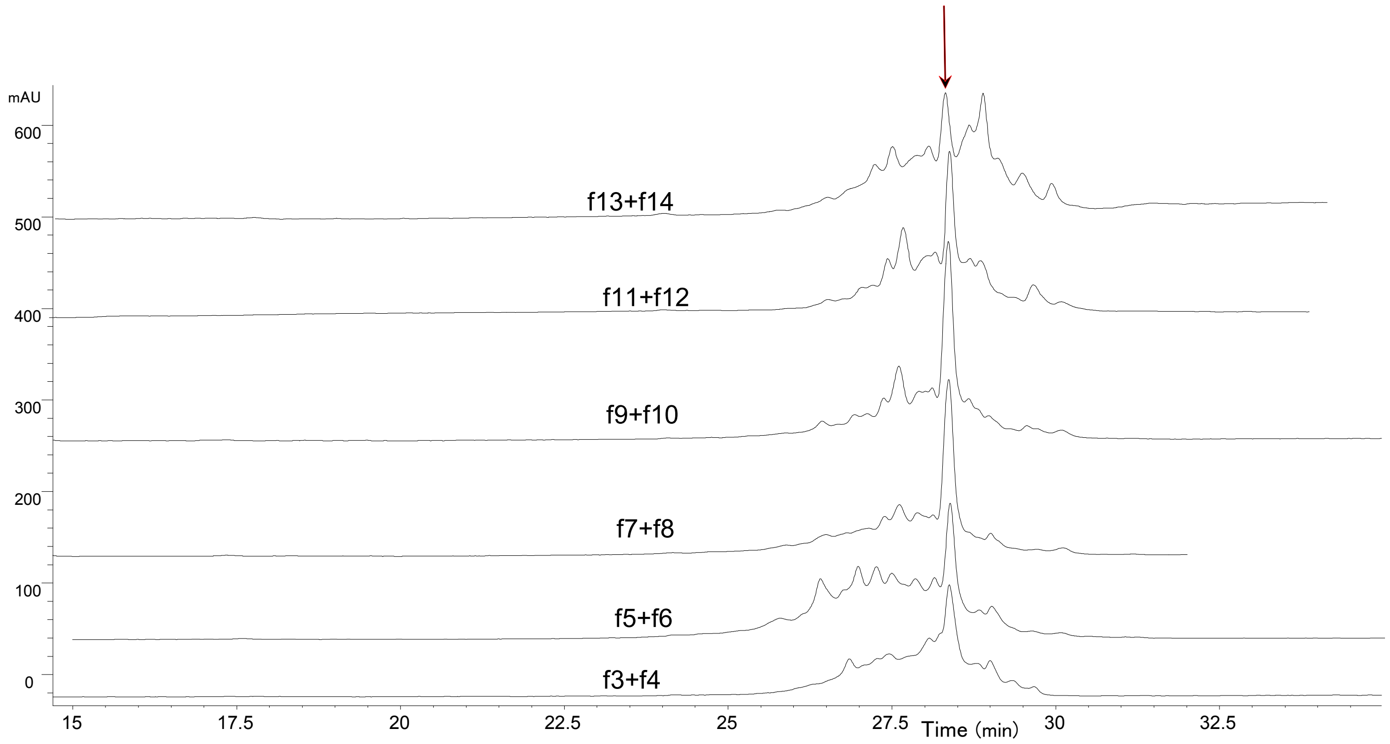

Figure S8. Injection of the fractions selected in the last step for purification of F44 in semi-preparative ion pair chromatography.

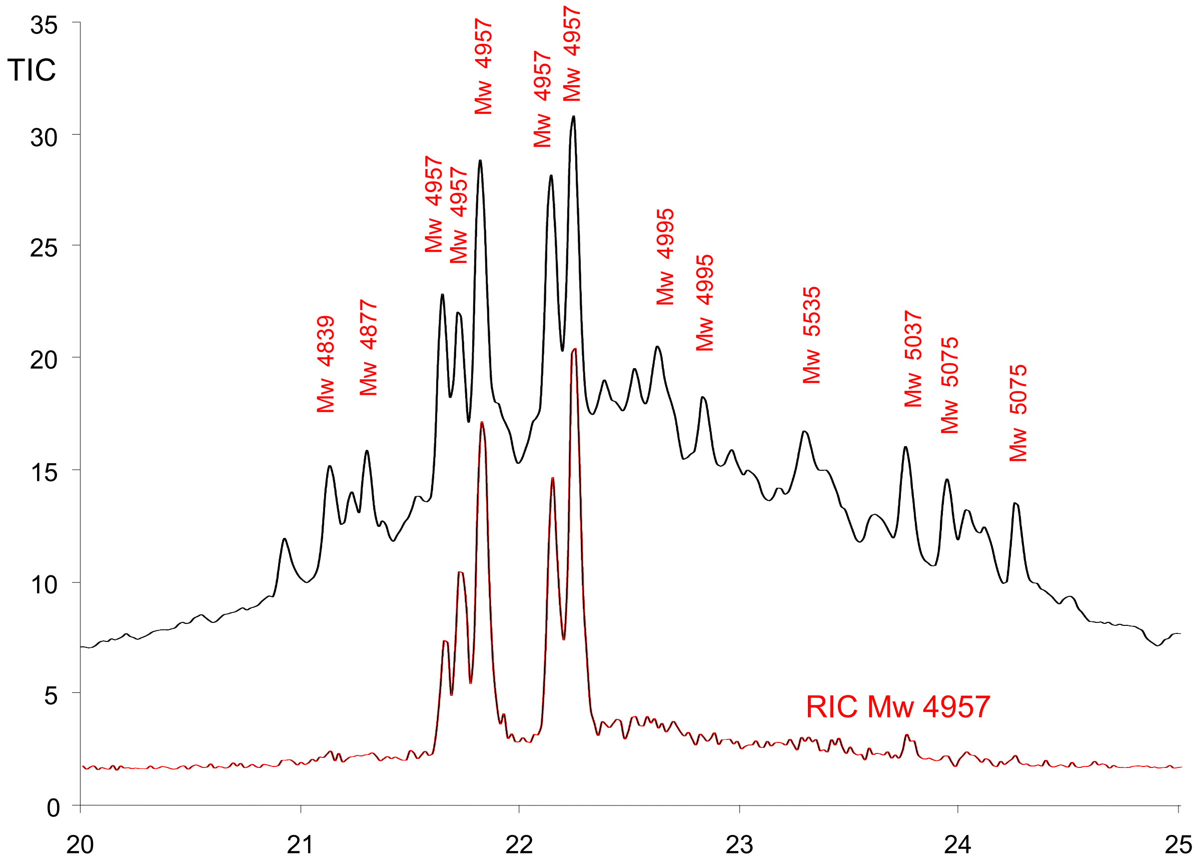

Figure S9. LC/MS of fraction F49.

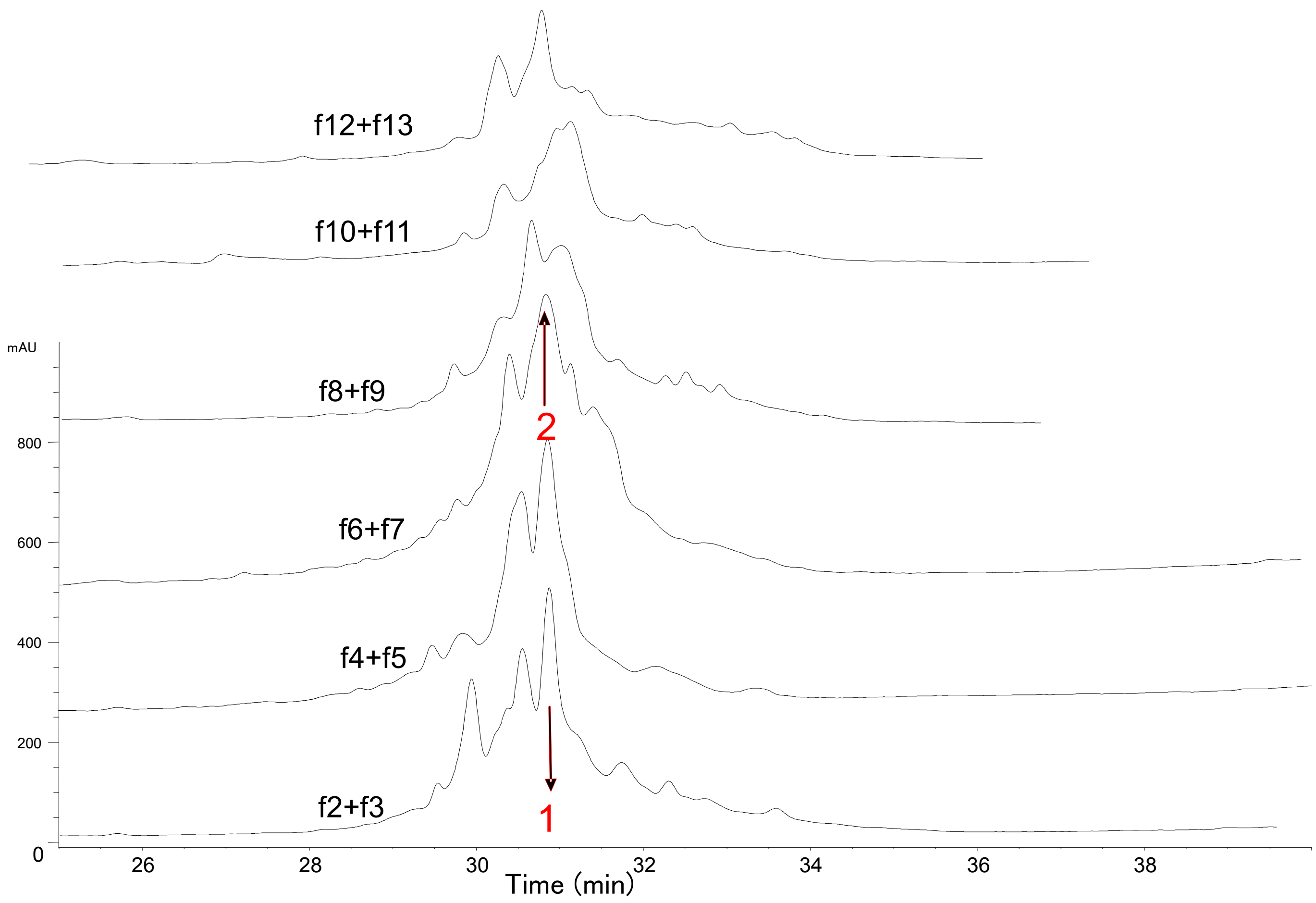

Figure S10. Injection of the fractions selected in the last step for purification of F49 in semi-preparative ion pair chromatography.

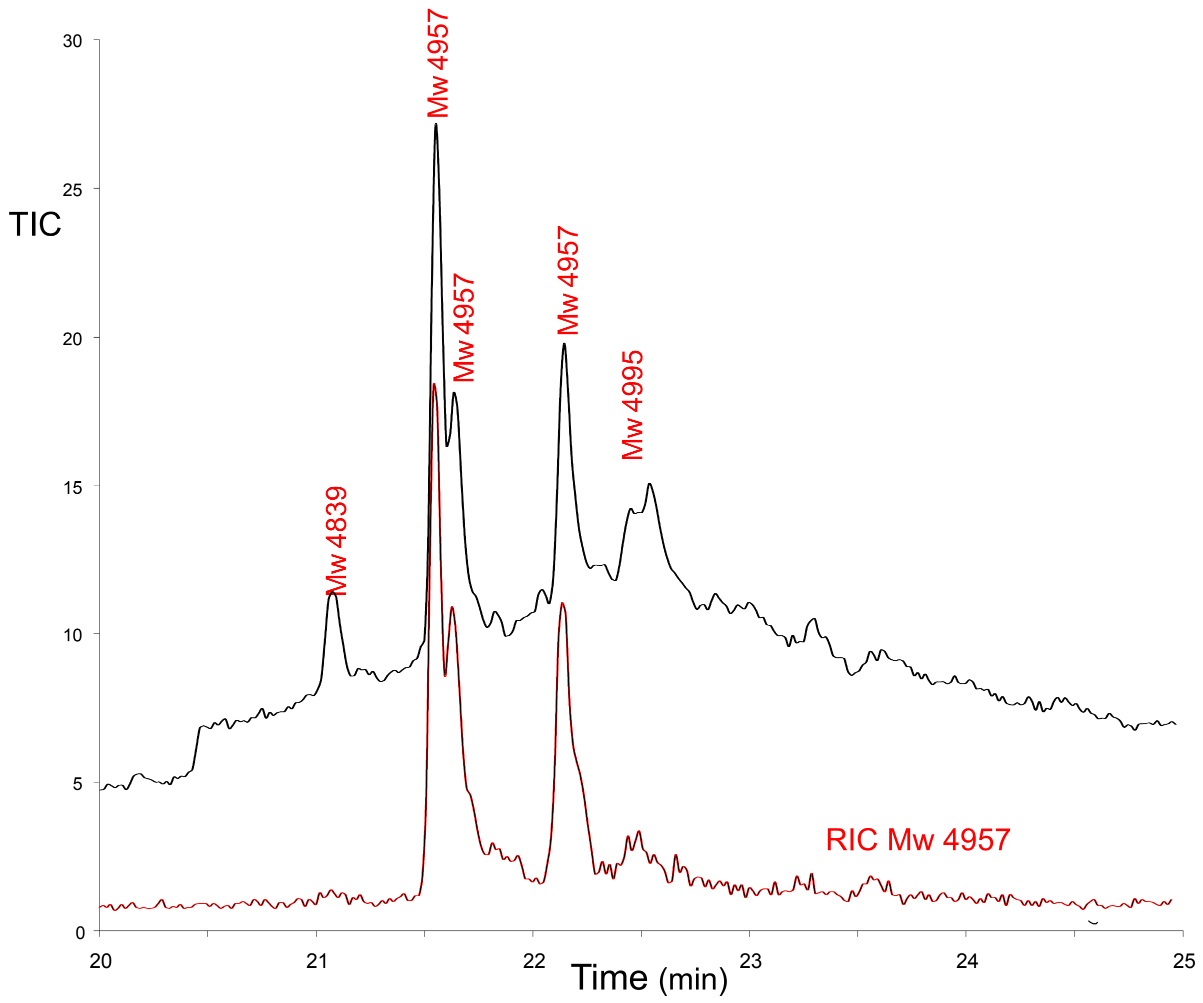

Figure S11. LC/MS of fraction F50.

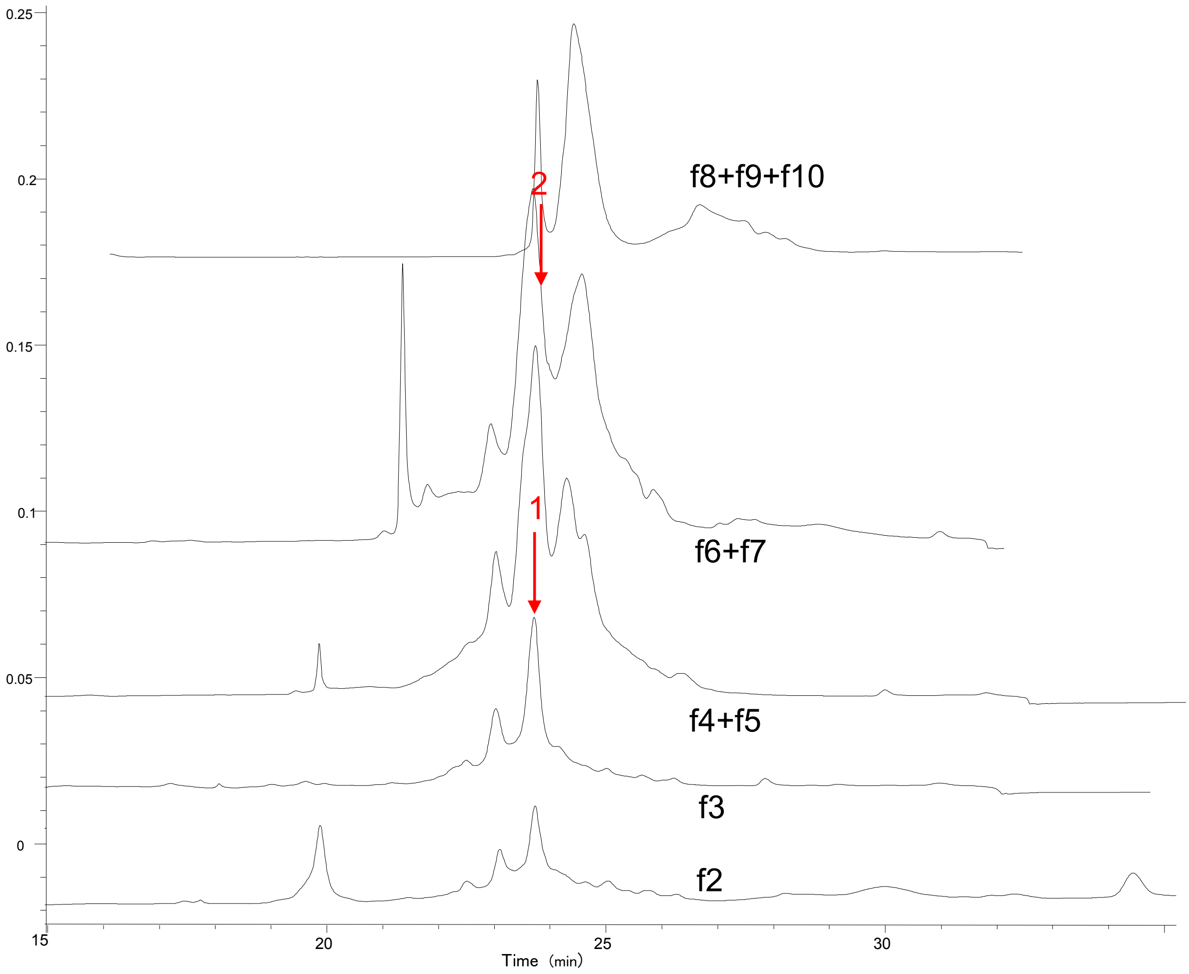

Figure S12. Injection of the fractions selected in the last step for purification of F50 in semi-preparative ion pair chromatography.

Sequencing experiments

Octadecasaccharide **2**

Figure S13 shows the CTA-SAX chromatogram of the purified octadecasaccharide **2**. LC/MS of the same compound is shown in Figure S14. The molecular weight 4957 Da is confirmed. The AS11 and LC/MS chromatograms of the heparinase I digestion products are shown in Figures S15 and S16. The observed fragmentation unambiguously conclude to the structure ΔIIa-$\underline{IIs_{glu}}$-Is$_{id}$-IIa$_{id}$-$\underline{IIs_{glu}}$-Is$_{id}$-Is$_{id}$-Is$_{id}$-Is$_{id}$ for octadeca 2. ΔIIa-$\underline{IIs_{glu}}$-Is$_{id}$-IIa$_{id}$-$\underline{IIs_{glu}}$ is detected in CTA-SAX and AS11 chromatography. The same fragment was observed for triple site octadecasaccharide **1** [1] in conjunction with ΔIs-IIa$_{id}$-$\underline{IIs_{glu}}$-Is$_{id}^{red}$. Here, the molecular weight 2310 corresponding to ΔIs-Is$_{id}$-Is$_{id}$-Is$_{id}^{red}$ is detected in LC/MS. Interestingly, the mass 2248 Da corresponding to ΔIIa-$\underline{IIs_{glu}}$-Is$_{id}$-IIa$_{id}$-GlcA is also observed in LC/MS. This is due to the partial alkaline cleavage of the terminal 3-O sulfated glucosamine (loss of 401 Da) occurring for ΔIIa-$\underline{IIs_{glu}}$-Is$_{id}$-IIa$_{id}$-$\underline{IIs_{glu}}$ in chromatographic conditions. This phenomenon was almost eliminated by lowering the column temperature from 40 °C to 30 °C. However, this 401 Da mass loss may be also a useful tool for structural

elucidation as it is a fingerprint of an oligosaccharide initially bearing a 3-O sulfated glucosamine at the reducing end.

Octadecasaccharide **3**

Figures S17 and S18 show the CTA-SAX and the LC/MS chromatograms of the purified octadecasaccharide **3**. Figures S19 and S20 show AS11 and LC/MS chromatograms of the heparinase I depolymerised octadecasaccharide **3** in the reduced form, and as it is, before $NaBH_4$ treatment (ΔIIa-$\underline{IIs_{glu}}$-Is_{id}-Is_{id}-IIa_{id}-$\underline{IIs_{glu}}$-Is_{id}-Is_{id}-Is_{id} structure). The fragmentation differences are directly linked to the decrease of heparinase I reactivity at the modified reducing end. More precisely, the reduced octadecasaccharide bears four cleavable sites (ΔIIa-$\underline{IIs_{glu}}$ $\overset{\downarrow}{-}$ Is_{id} $\overset{\downarrow}{-}$ Is_{id}-IIa_{id}-$\underline{IIs_{glu}}$ $\overset{\downarrow}{-}$ Is_{id} $\overset{\downarrow}{-}$ Is_{id}-Is_{id}^{red}) whereas, five depolymerisation possibilities exists in its endogenous form (ΔIIa-$\underline{IIs_{glu}}$ $\overset{\downarrow}{-}$ Is_{id} $\overset{\downarrow}{-}$ Is_{id}-IIa_{id}-$\underline{IIs_{glu}}$ $\overset{\downarrow}{-}$ Is_{id} $\overset{\downarrow}{-}$ Is_{id} $\overset{\downarrow}{-}$ Is_{id}). In addition, the reduction influences the depolymerisation behavior of the three or four next disaccharides. The fragmentation of the non-reducing side is identical for the two oxidation states of the substrate. The key fragment ΔIIa-$\underline{IIs_{glu}}$-Is_{id}-Is_{id}-IIa_{id}-$\underline{IIs_{glu}}$ is detected on both chromatograms. ΔIs-Is_{id}-IIa_{id}-$\underline{IIs_{glu}}$, fragment corresponding to the middle of the chain, is detected in the two cases (ΔIs-Is_{id}-IIa_{id}-GlcA degradation product (1789 Da = 2190-401) is also observed in LC/MS, giving the confirmation of the 3-O sulfated glucosamine at the reducing end). Fragments corresponding to the cleavages on the reducing side are different for the two oxidation states. For the reduced octadecasaccharide , ΔIs-Is_{id}^{red}, ΔIs-Is_{id}-Is_{id}^{red} and ΔIIa-$\underline{IIs_{glu}}$-Is_{id}-Is_{id}-IIa_{id}-$\underline{IIs_{glu}}$-Is_{id} are present. For the endogenous compound, two major fragments at 3344 Da and 3920 Da corresponding respectively to ΔIs-IIa_{id}-$\underline{IIs_{glu}}$-Is_{id}-Is_{id}-Is_{id} and ΔIs-Is_{id}-IIa_{id}-$\underline{IIs_{glu}}$-Is_{id}-Is_{id}-Is_{id} are observed.

Octadecasaccharide **4**

Figure S21 shows the CTA-SAX chromatogram of the purified octadecasaccharide **4**. Figure S22 shows its LC/MS chromatogram, confirming the molecular weight at 4957 Da. The AS11 and LC/MS chromatograms of the heparinase digests are shown in Figures S23 and S24. The key fragments ΔIIs-IIa_{id}-$\underline{IIs_{glu}}$, ΔIIs-IIa_{id}-$\underline{IIs_{glu}}$-Is_{id}-Is_{id}-IIa_{id}-$\underline{IIs_{glu}}$ and ΔIs-Is_{id}^{red} were detected in CTA-SAX and their molecular weight confirmed by LC/MS. In the LC/MS experiment, concomitantly with the fragment at 4723 Da corresponding to ΔIIs-IIa_{id}-$\underline{IIs_{glu}}$-Is_{id}-Is_{id}-IIa_{id}-$\underline{IIs_{glu}}$, one degradation fragment at 4322 Da (-401 Da: loss of the terminal GlcNS6S3S) was also observed. ΔIIs-IIa_{id}-$\underline{IIs_{glu}}$-Is_{id}-Is_{id}-IIa_{id}-$\underline{IIs_{glu}}$ is rapidly depolymerized into a mixture of ΔIIs-IIa_{id}-$\underline{IIs_{glu}}$, ΔIs-Is_{id}-IIa_{id}-$\underline{IIs_{glu}}$ and ΔIs-IIa_{id}-$\underline{IIs_{glu}}$. The presence of ΔIs-Is_{id}-IIa_{id}-$\underline{IIs_{glu}}$ is confirmed in LC/MS by the -401 Da fragment (1789 Da) corresponding to ΔIs-Is_{id}-IIa_{id}-GlcA.

Octadecasaccharide **5**

Figures S25 and S26 show the CTA-SAX and LC/MS chromatograms of the purified octadecasaccharide **5**. In Figure S26, the saturation of the signal strongly magnified the detection of a second minor component (ΔIs-IIa_{id}-$\underline{IIs_{glu}}$-Is_{id}-IIa_{id}-$\underline{IIs_{glu}}$-Is_{id}-Is_{id}-Is_{id}). Heparinase I digests chromatographed on AS11 and LC/MS are shown in Figures S27 and S28. Key fragments ΔIIs-IIa_{id}-IIs_{glu} and ΔIs-IIa_{id}-IIs_{glu}-Is_{id}^{red} are easily detected on both techniques. Additionally, LC/MS detects fragments at 3346 Da and 2789 Da corresponding to the reducing part of the chain, respectively ΔIs-Is_{id}-Is_{id}-IIa_{id}-$\underline{IIs_{glu}}$-Is_{id}^{red} and ΔIs-Is_{id}-IIa_{id}-$\underline{IIs_{glu}}$-Is_{id}^{red}. The expected structure ΔIs-IIa_{id}-$\underline{IIs_{glu}}$-Is_{id}-Is_{id}-Is_{id}-IIa_{id}-$\underline{IIs_{glu}}$-Is_{id} is confirmed.

Octadecasaccharide **6**

Figures S29 and S30 show the CTA-SAX and LC/MS chromatograms of the purified octadecasaccharide **6**. The minor peak detected on both techniques (CTA-SAX: 48 min) and LC/MS 4719 Da is due to the partial loss of the unsaturated acid ΔGlcA2S (-238 Da). Figures S31 and S32 show the chromatograms with AS11 column and ion pair LC/MS of the heparinase I sequencing experiments. In Figure S31, chromatograms reflecting three different reaction time points show the kinetics of enzymatic digestion and key fragments. The fragment ΔIIs-Is$_{id}$-Is$_{id}$-IIa$_{id}$-IIs$_{glu}$, giving the position of the first AT binding site in the chain is rapidly depolymerised in a mixture of ΔIs-Is$_{id}$-IIa$_{id}$-IIs$_{glu}$, ΔIs-IIa$_{id}$-IIs$_{glu}$, ΔIs-Is$_{id}$, ΔIIs-Is$_{id}$ and ΔIIs. Its structural identification was confirmed by Δ-2-O desulfatation of ΔIs-Is$_{id}$-Is$_{id}$-IIa$_{id}$-IIs$_{glu}$ in a decasaccharide fraction of heparinase 1 depolymerised heparin (not shown). In the LC/MS chromatogram, other fragments of the non-reducing side such as GlcNS6S-Is$_{id}$-Is$_{id}$-IIa$_{id}$-IIs$_{glu}$ (Mw 2529 Da) and ΔIIs-Is$_{id}$-Is$_{id}$-IIa$_{id}$-GlcA (Mw 2286 Da) were detected. The other key fragment ΔIs-IIa$_{id}$-IIs$_{glu}$-Is$_{id}^{red}$ of the reducing side is obviously much easier to detect. The characteristic main fragments confirm the ΔIs-Is$_{id}$-Is$_{id}$-IIa$_{id}$-IIs$_{glu}$-Is$_{id}$-IIa$_{id}$-IIs$_{glu}$-Is$_{id}$ structure for octadecasaccharide **6**.

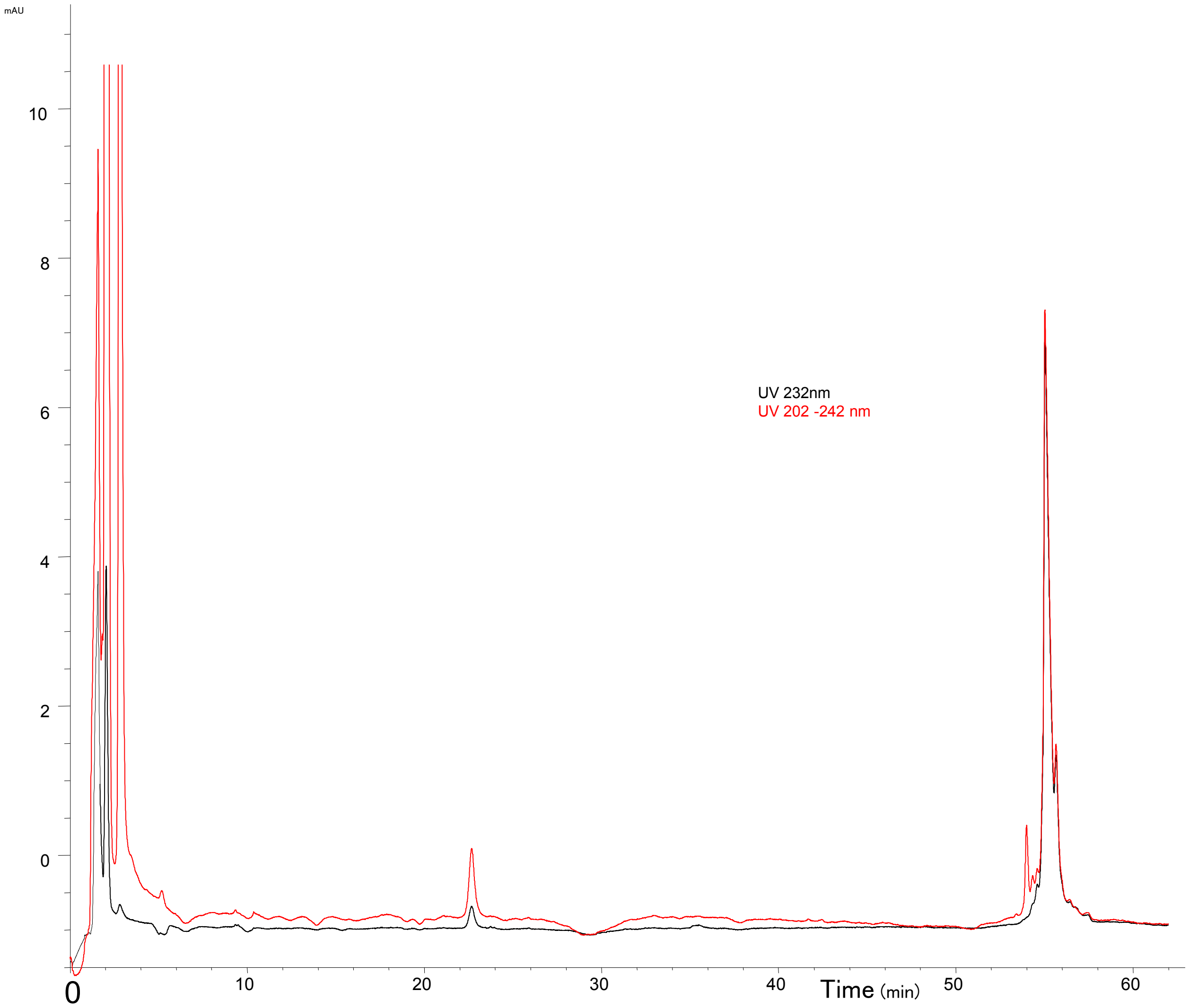

Figure S13. CTA-SAX chromatogram of the purified octadecasaccharide **2.**

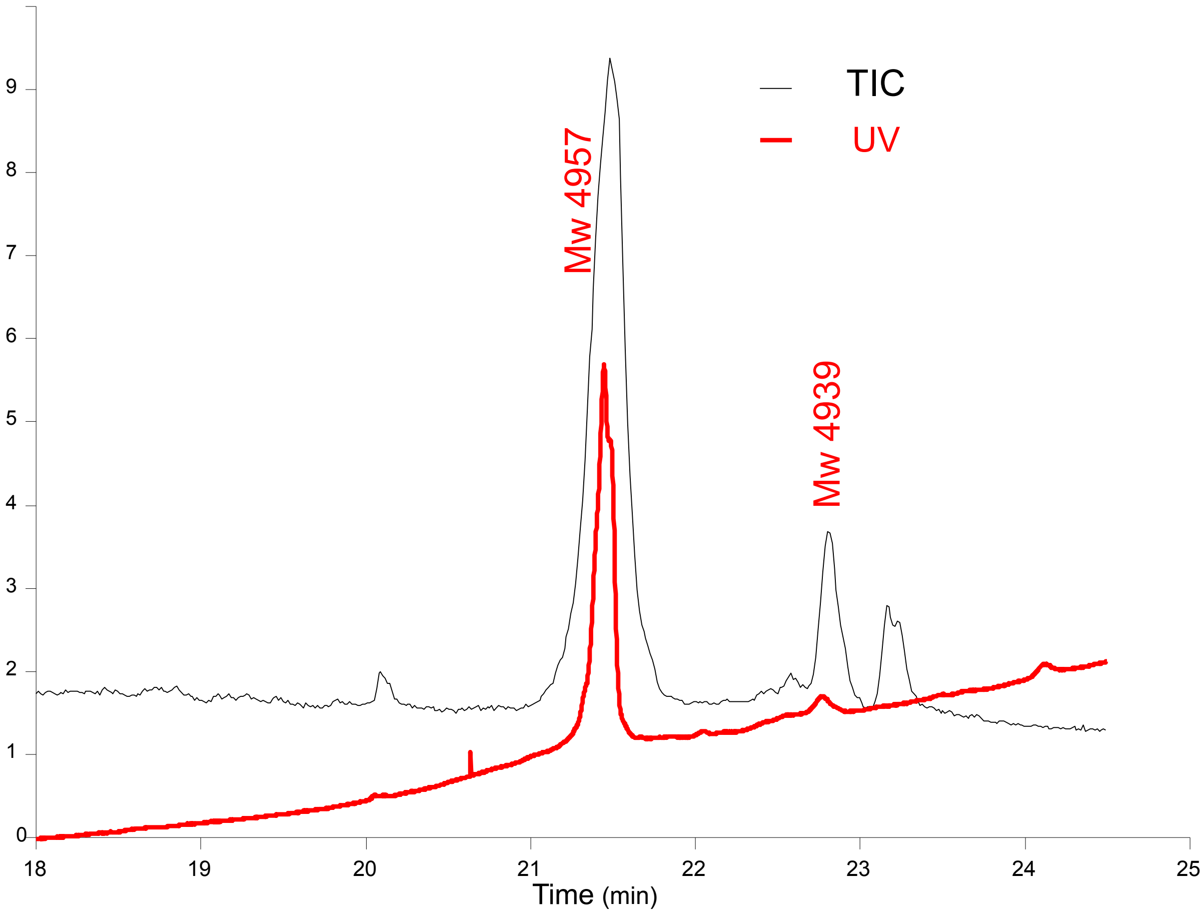

Figure S14. LC/MS chromatogram of the purified octadecasaccharide **2.**

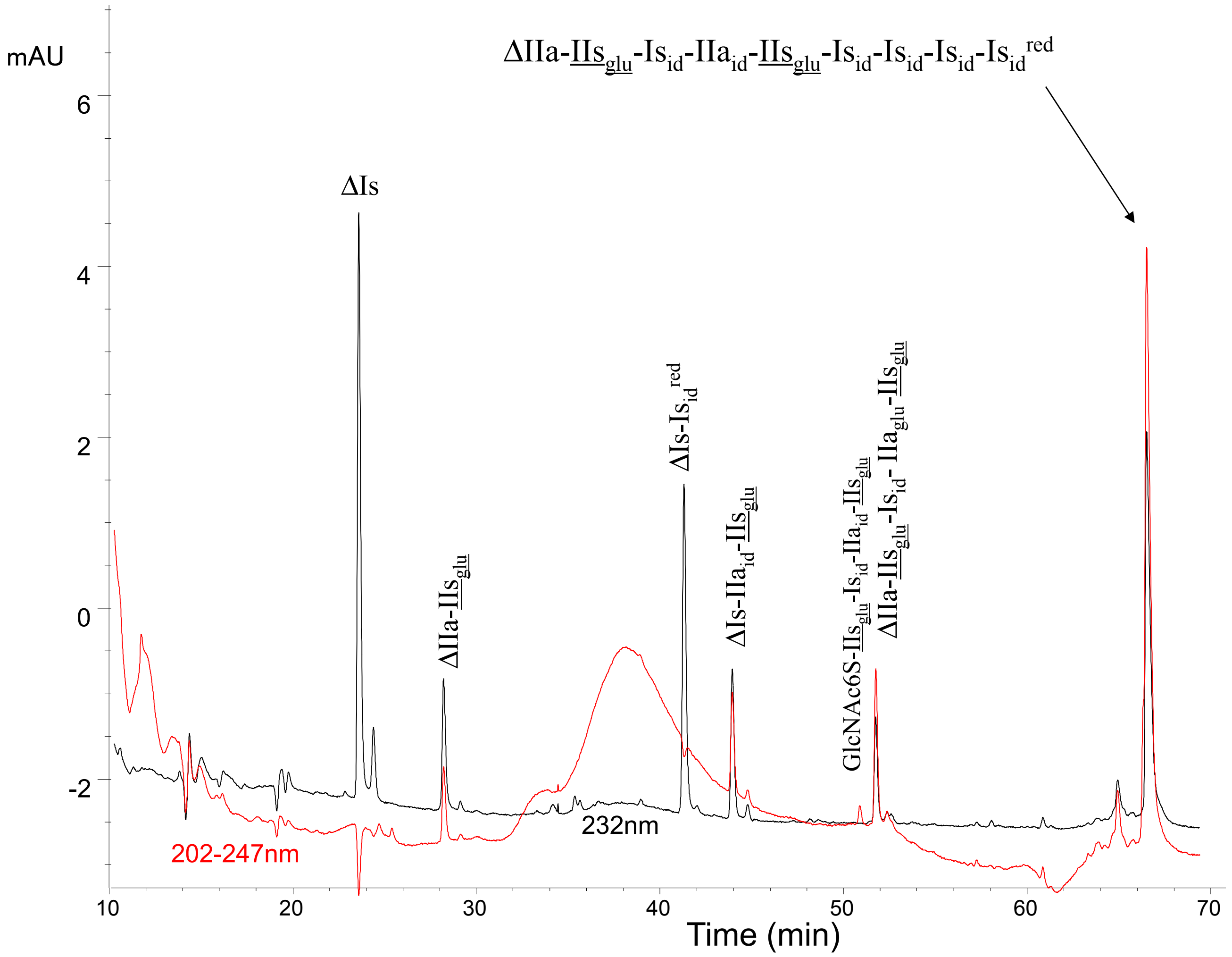

Figure S15. Sequencing of octadecasaccharide2, chromatographic follow-up on AS11 of the depolymerisation of octadecasaccharide **2** by heparinase I.

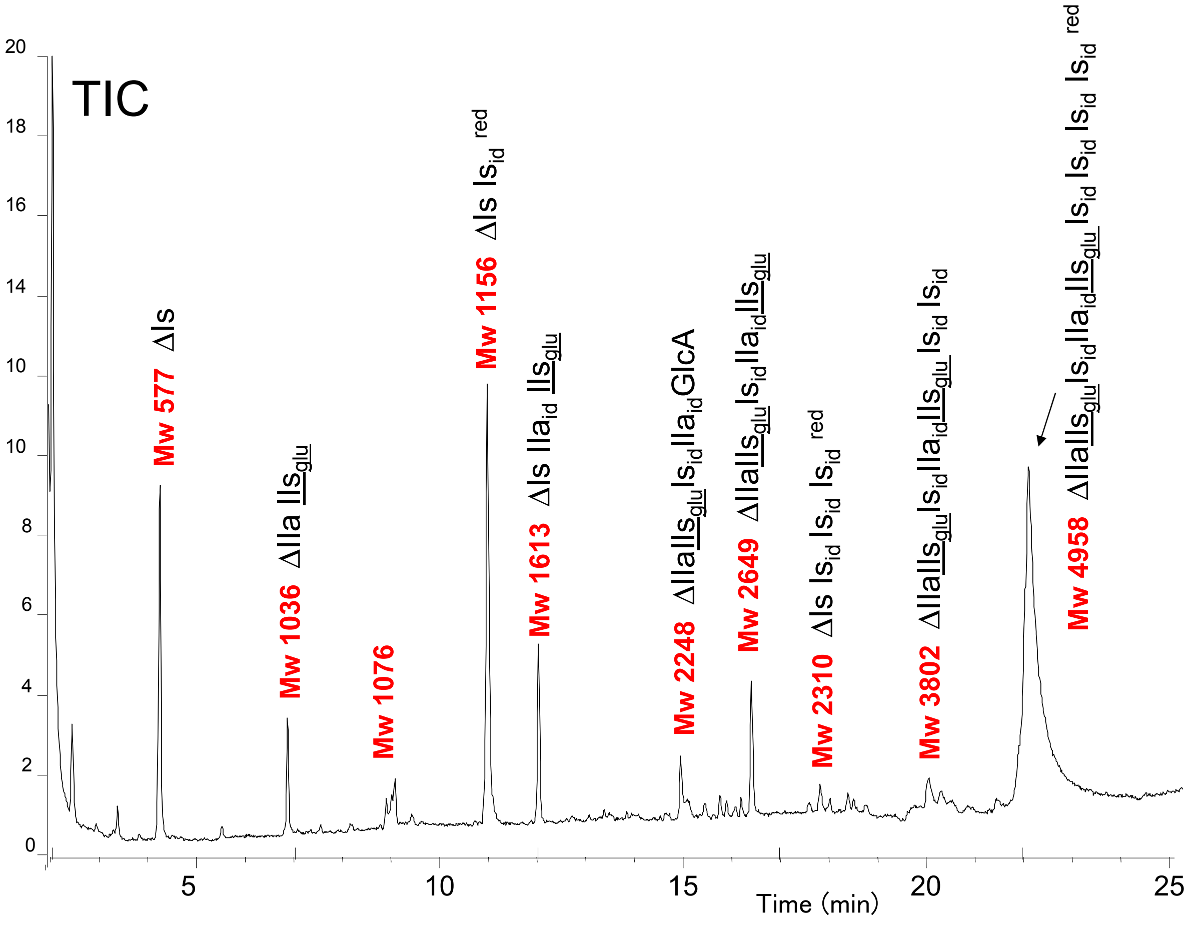

Figure S16. Sequencing of octadecasaccharide **2**, LC/MS follow-up of the depolymerisation of octadecasaccharide **2** by heparinase I.

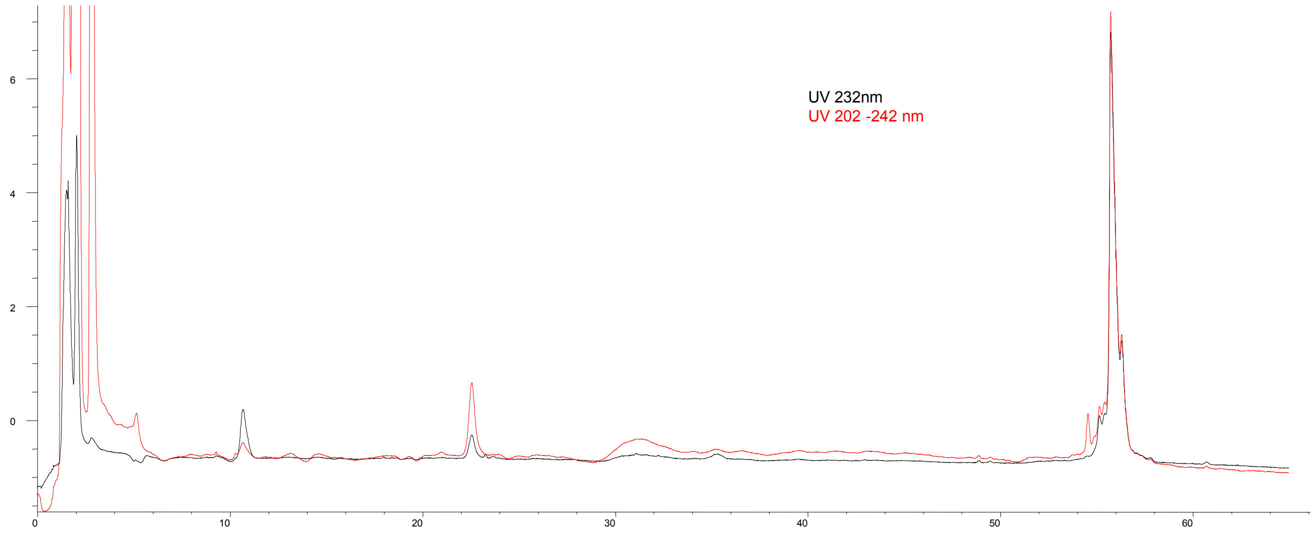

Figure S17. CTA-SAX chromatogram of the purified octadecasaccharide **3.**

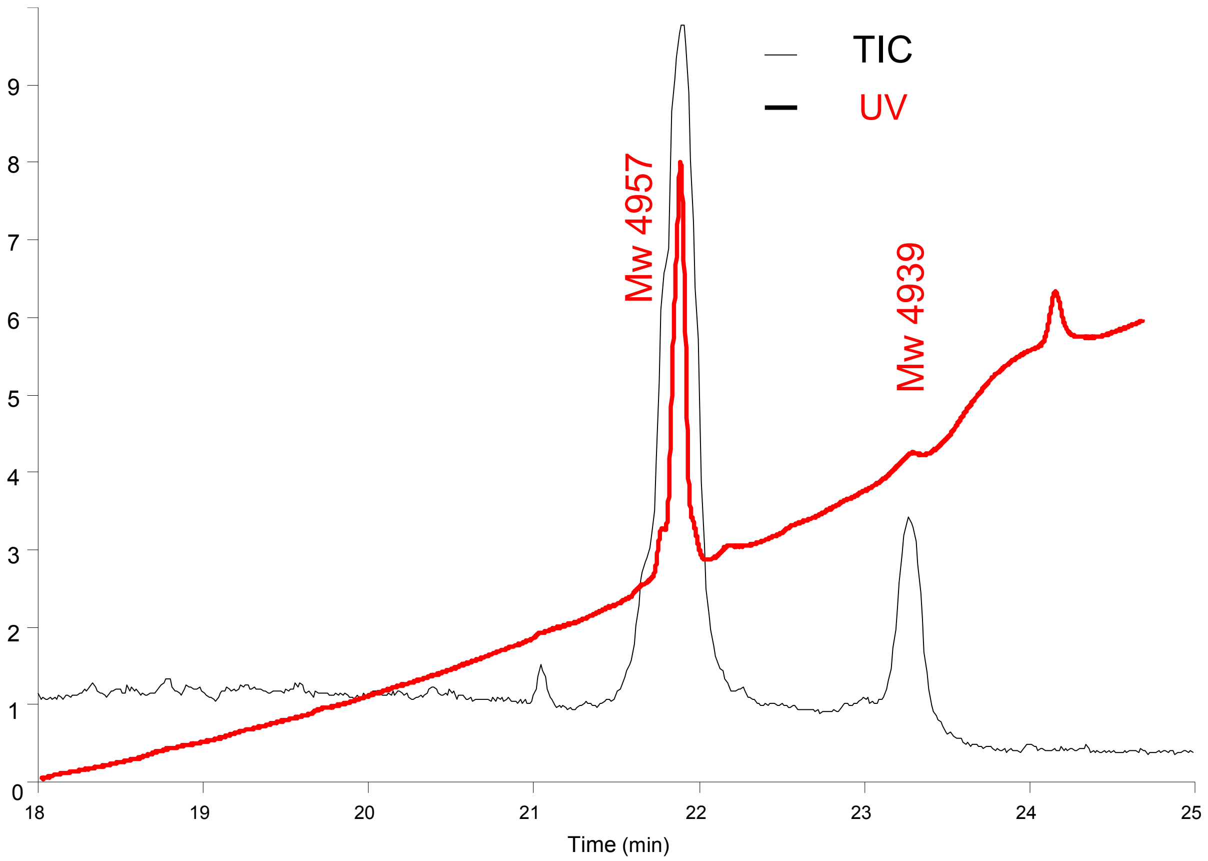

Figure S18. LC/MS of the purified octadecasaccharide **3.**

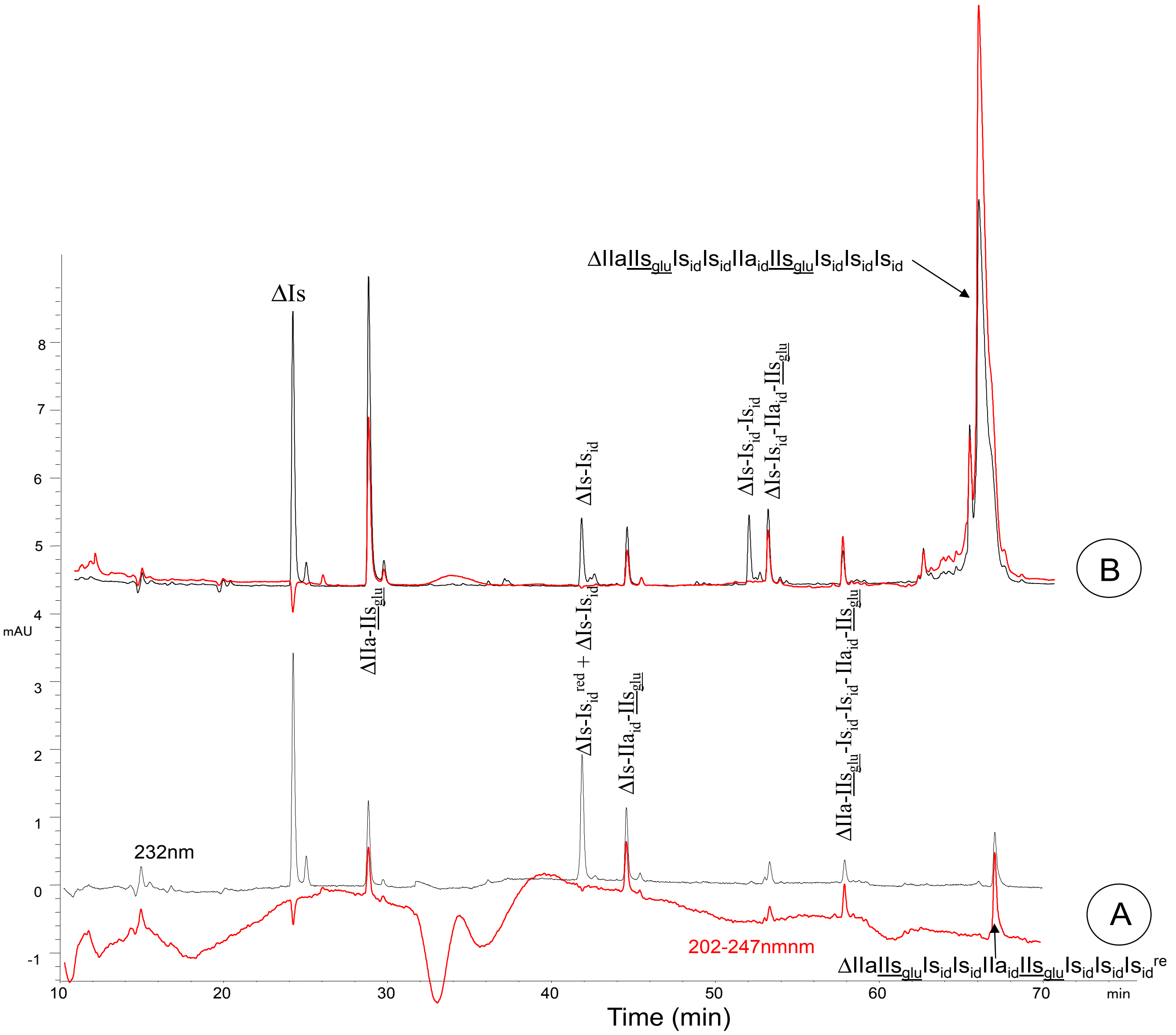

Figure S19. Sequencing of octadecasaccharide **3**, chromatographic follow-up on AS11 of the depolymerisation of octadecasaccharide **3** by heparinase I (reduced octadecasaccharide **3** (A) versus octadecasaccharide **3**).

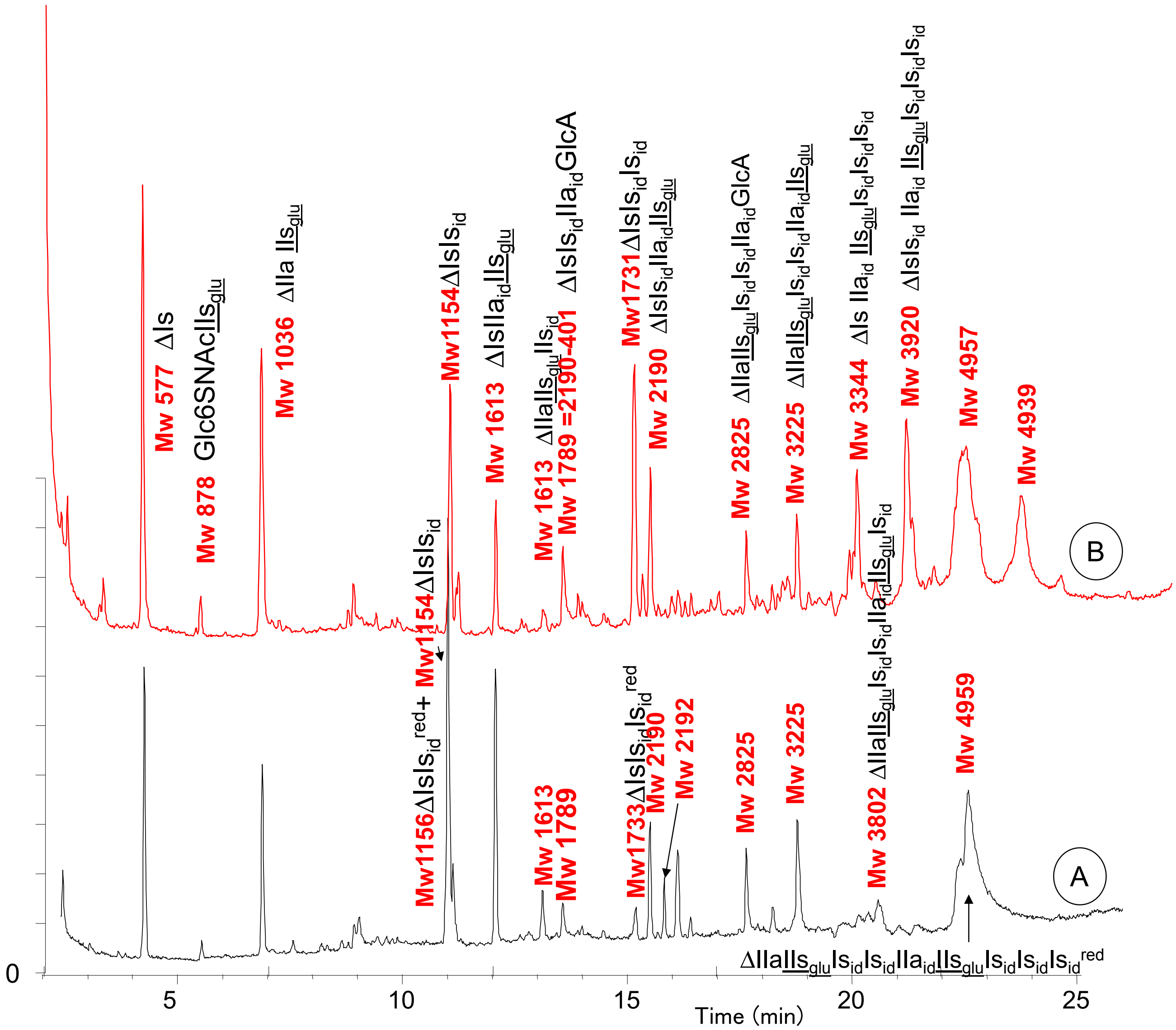

Figure S20. Sequencing of octadecasaccharide **3**: LC/MS follow-up of the depolymerisation of octadecasaccharide **3** by heparinase I (reduced octadecasaccharide **3** (A) versus octadecasaccharide **3** (B)).

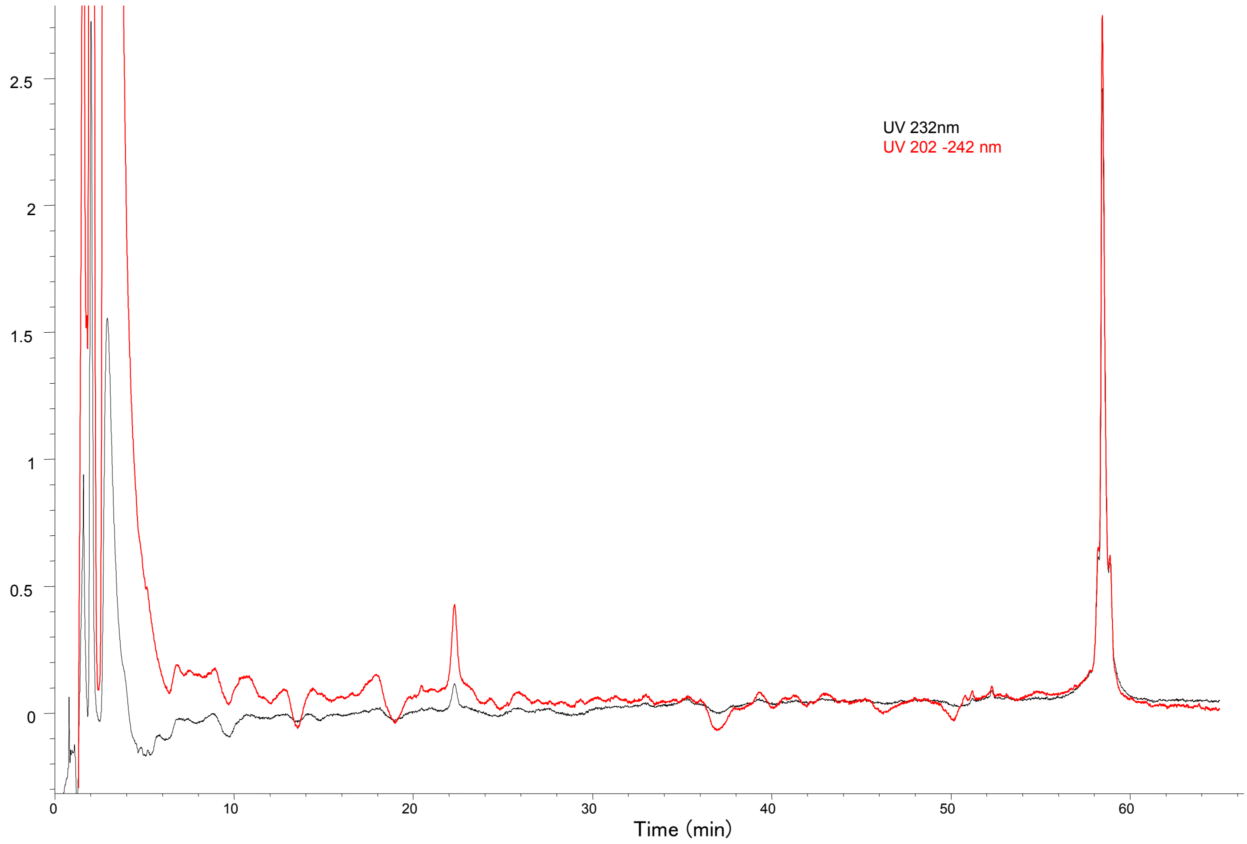

Figure S21. CTA-SAX chromatogram of the purified octadecasaccharide **4.**

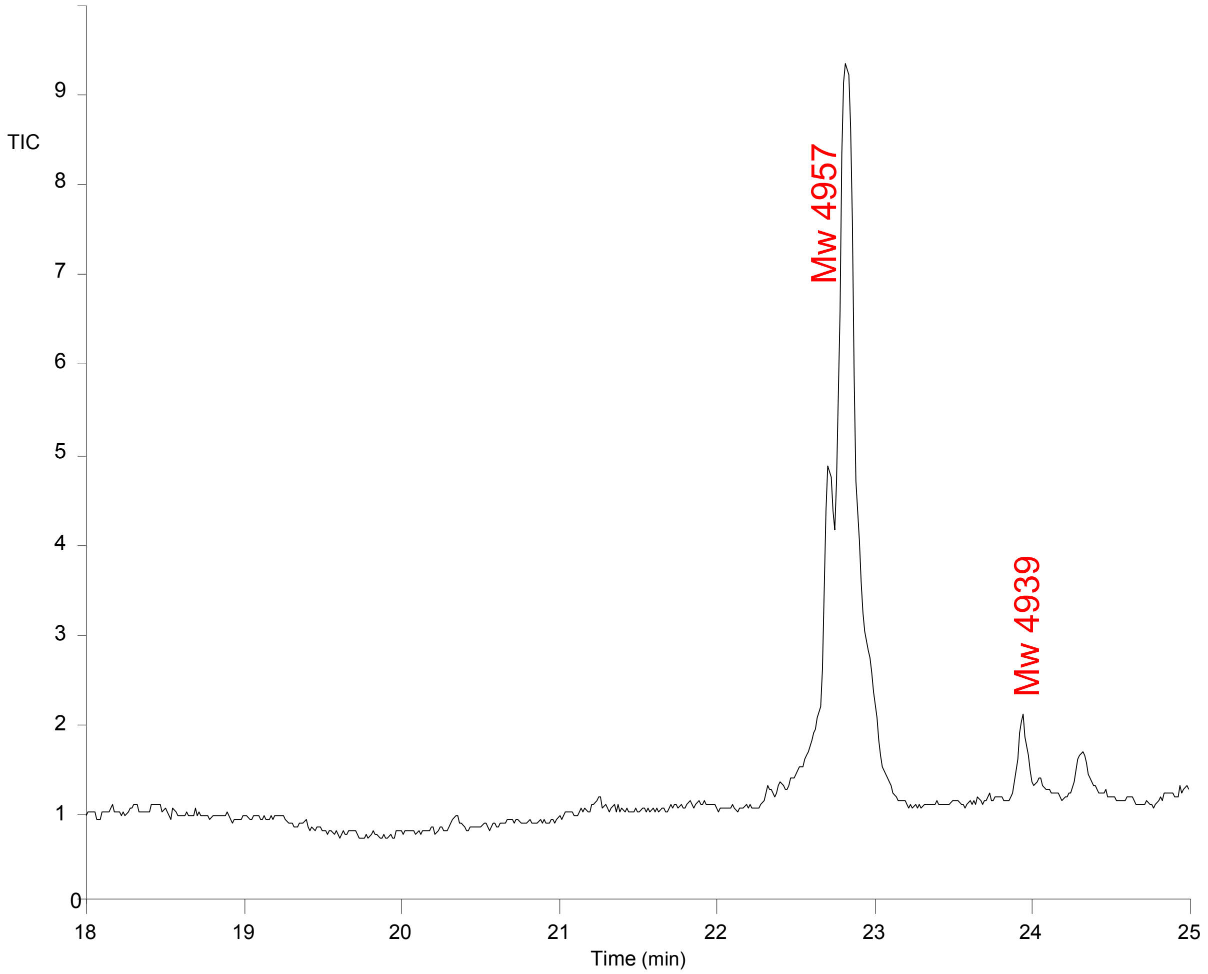

Figure S22. LC/MS chromatogram of the purified octadecasaccharide **4.**

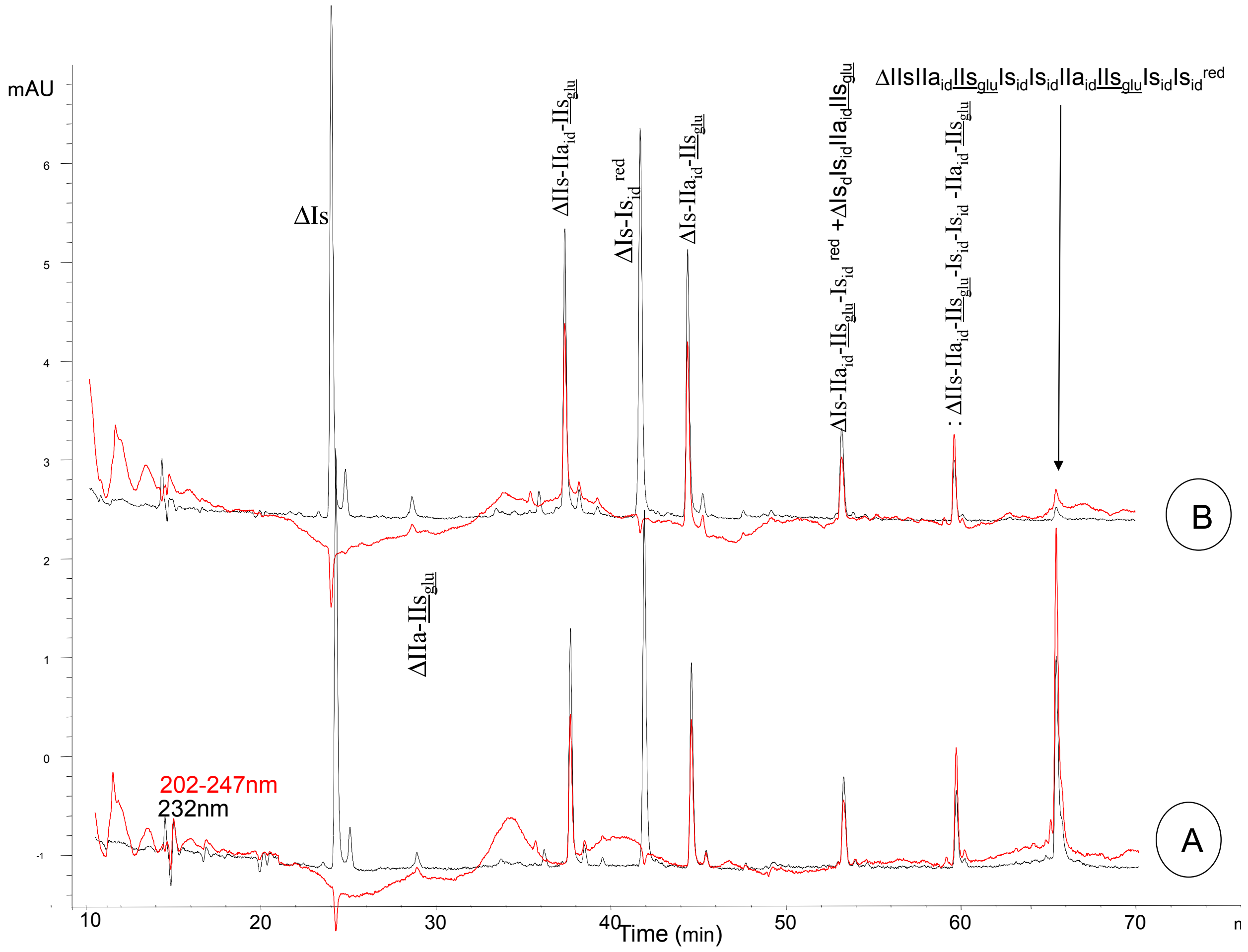

Figure S23. Sequencing of octadecasaccharide **4**, chromatographic follow-up on AS11 of the depolymerisation of octadecasaccharide **4** by heparinase I.

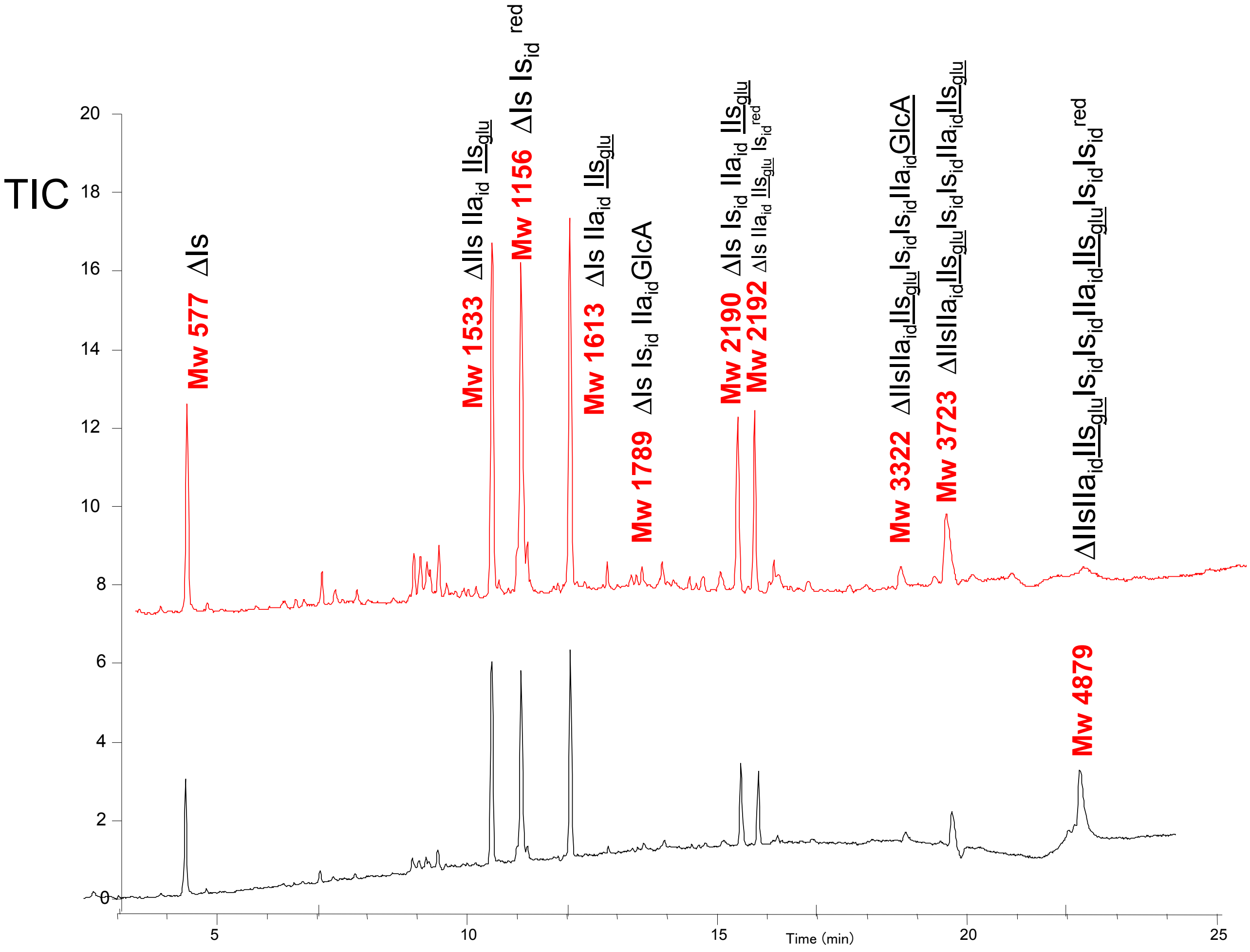

Figure S24. Sequencing of octadecasaccharide **4**, LC/MS follow-up of the depolymerisation of octadecasaccharide **4** by heparinase I.

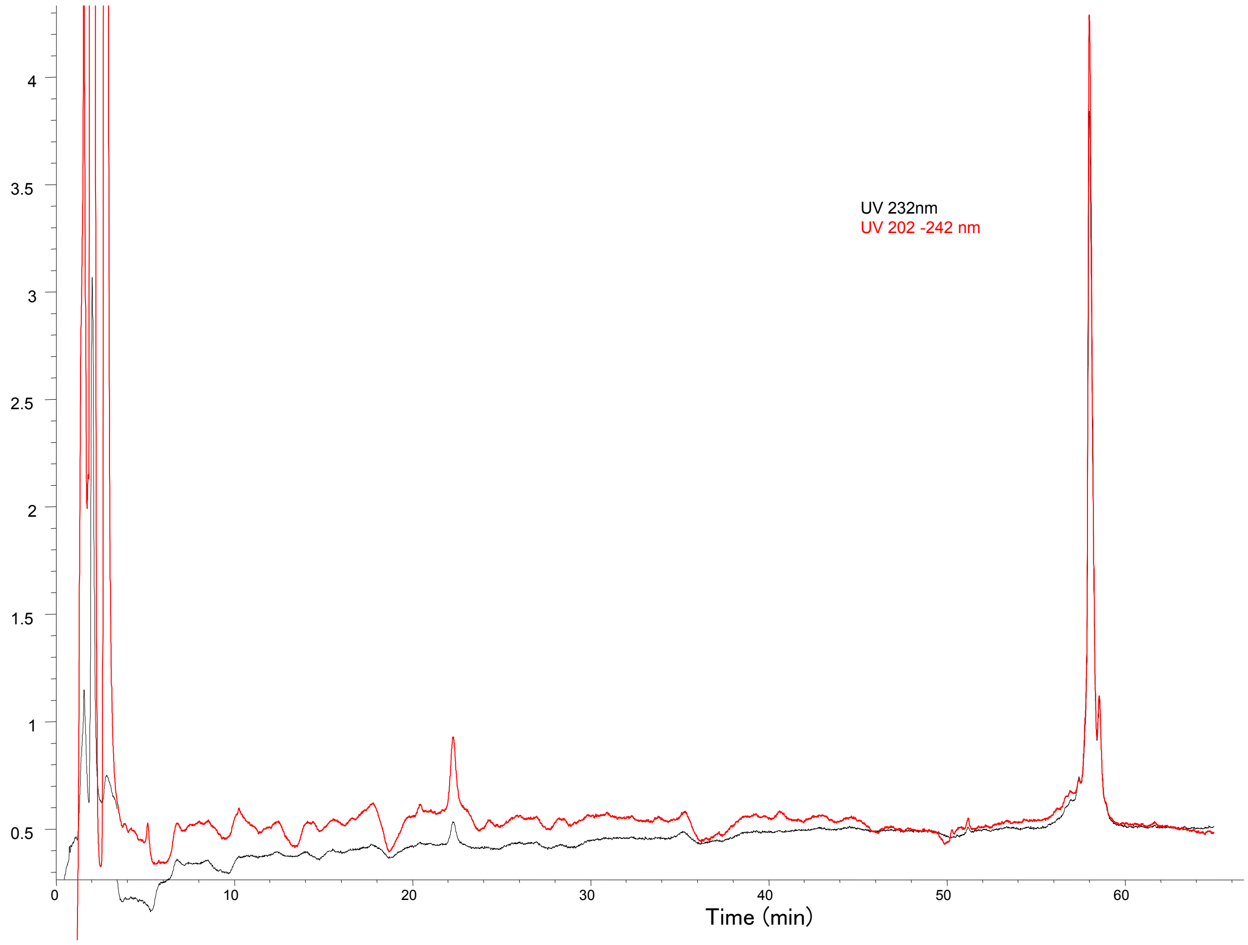

Figure S25. CTA-SAX chromatogram of the purified octadecasaccharide **5.**

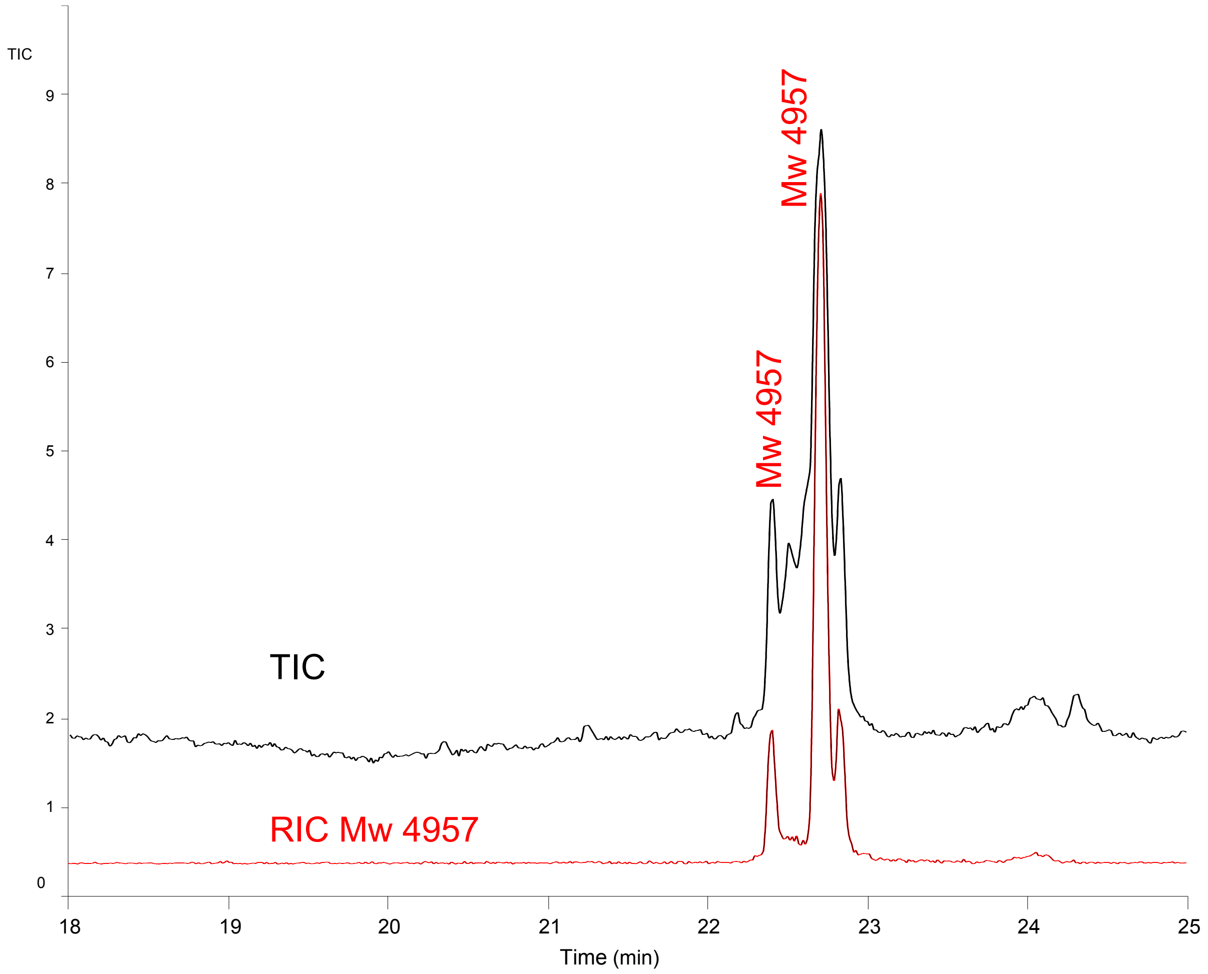

Figure S26. LC/MS chromatogram of the purified octadecasaccharide **5.**

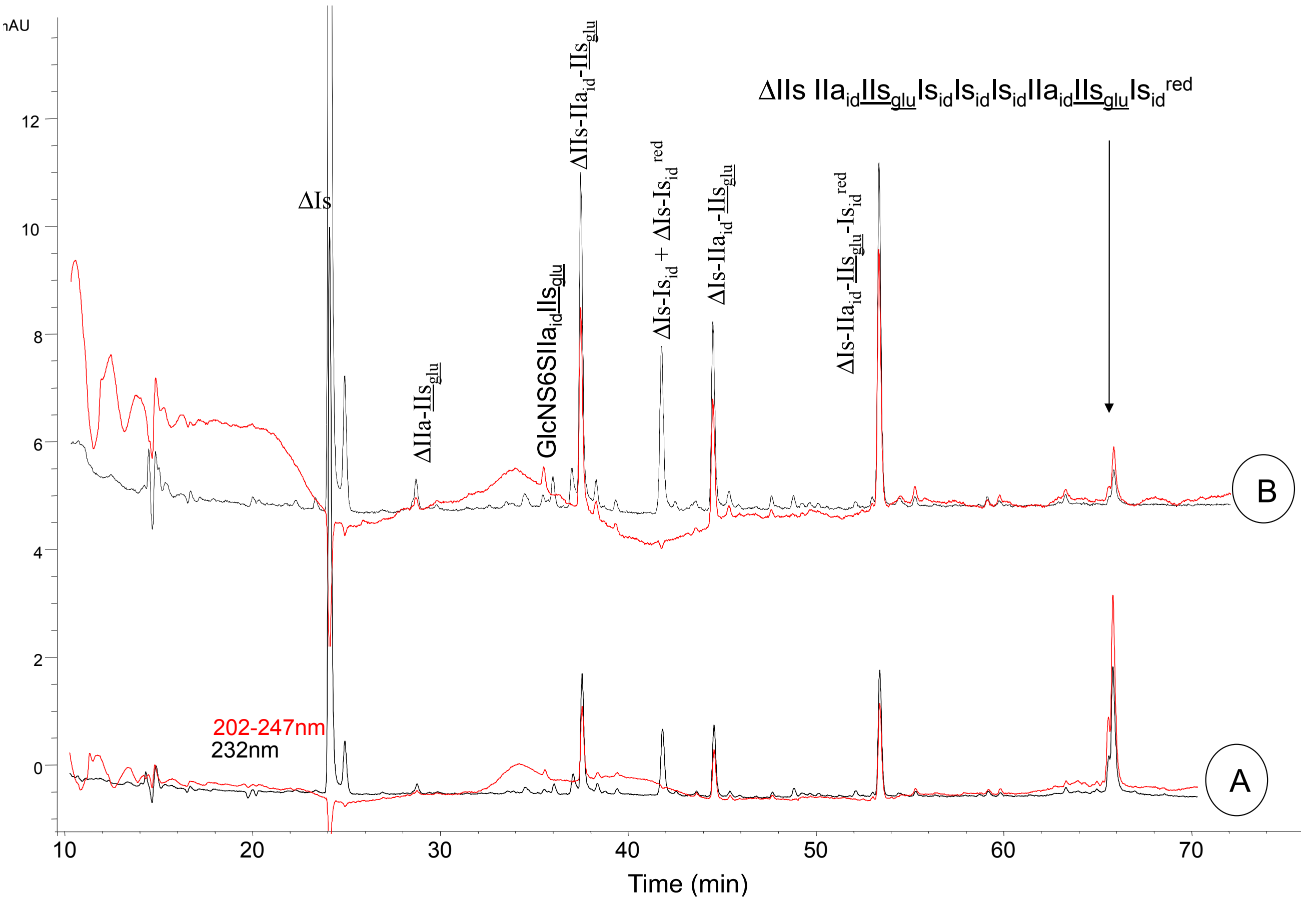

Figure S27. Sequencing of octadecasaccharide **5**, chromatographic follow-up on AS11 of the depolymerisation of octadecasaccharide **5** by heparinase I.

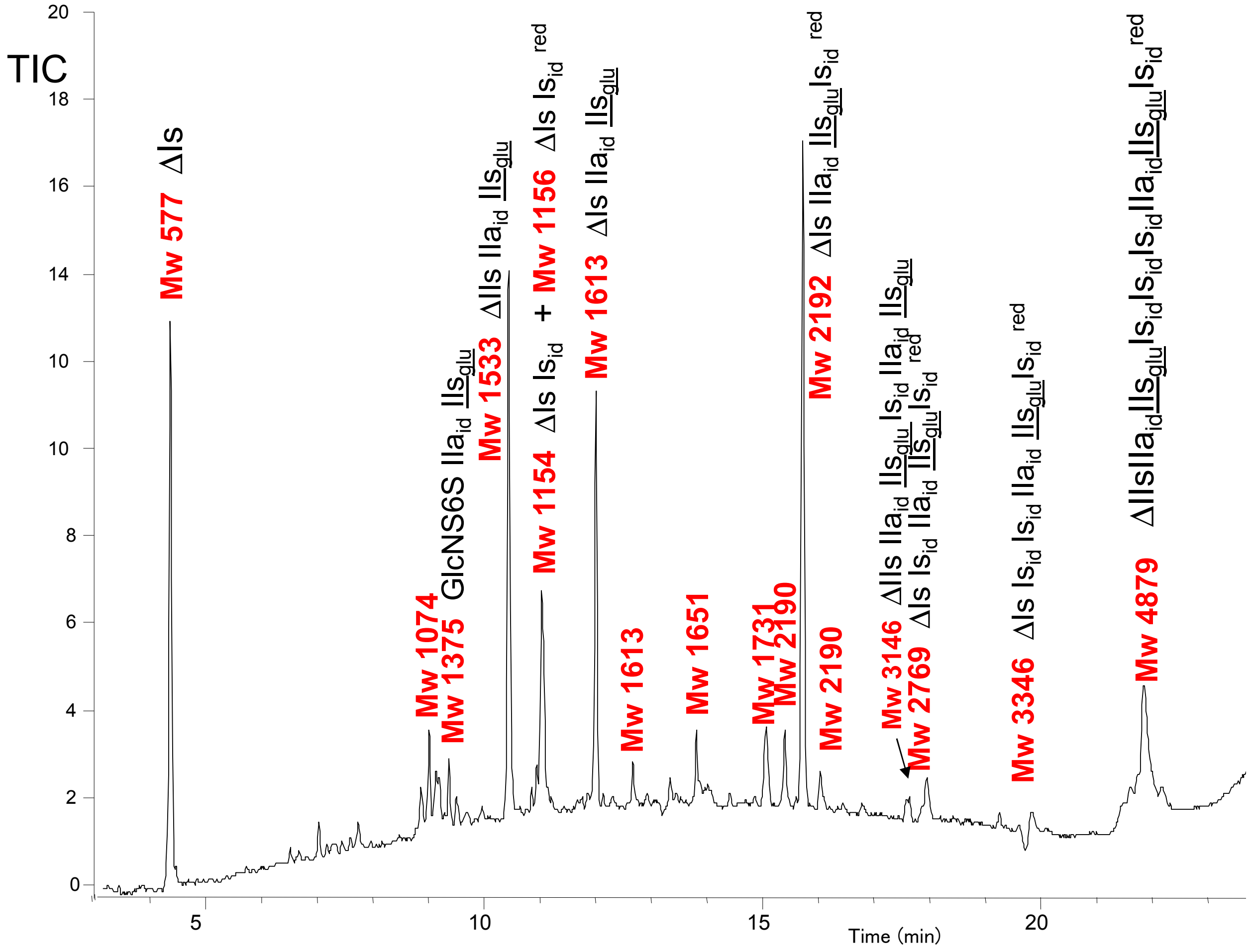

Figure S28. Sequencing of octadecasaccharide **5**, LC/MS follow-up of the depolymerisation of octadecasaccharide **5** by heparinase I.

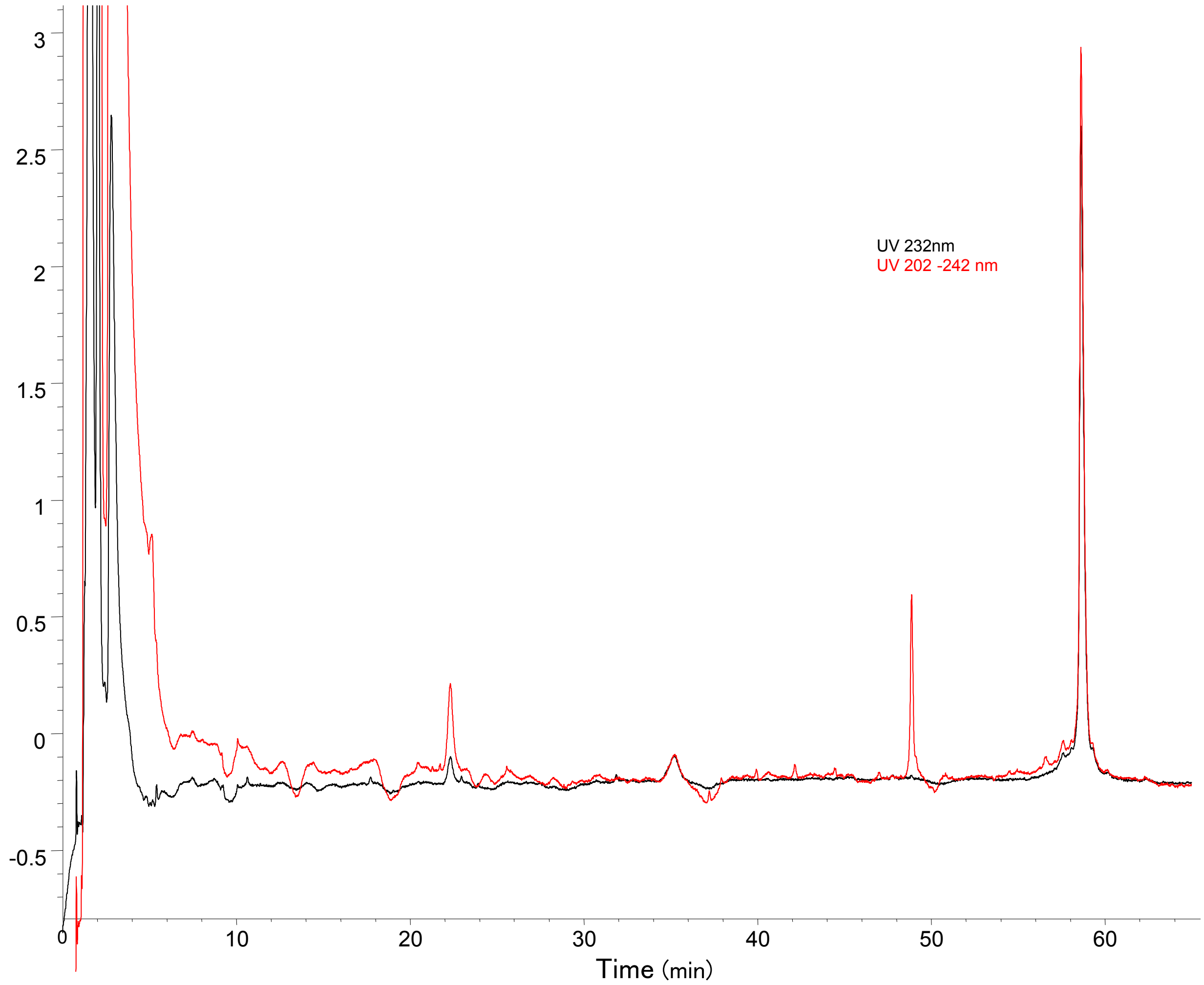

Figure S29. CTA-SAX chromatogram of the purified octadecasaccharide **6.**

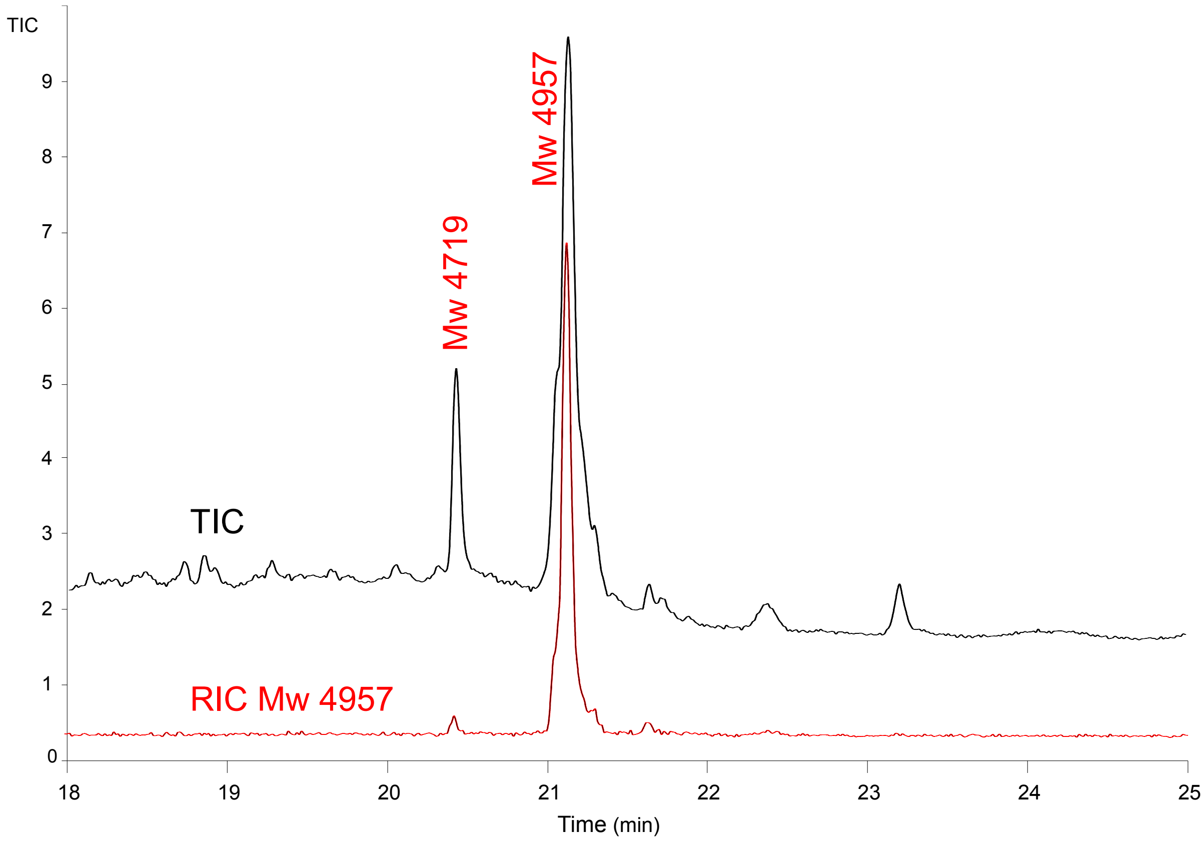

Figure S30. LC/MS of the purified octadecasaccharide **6.**

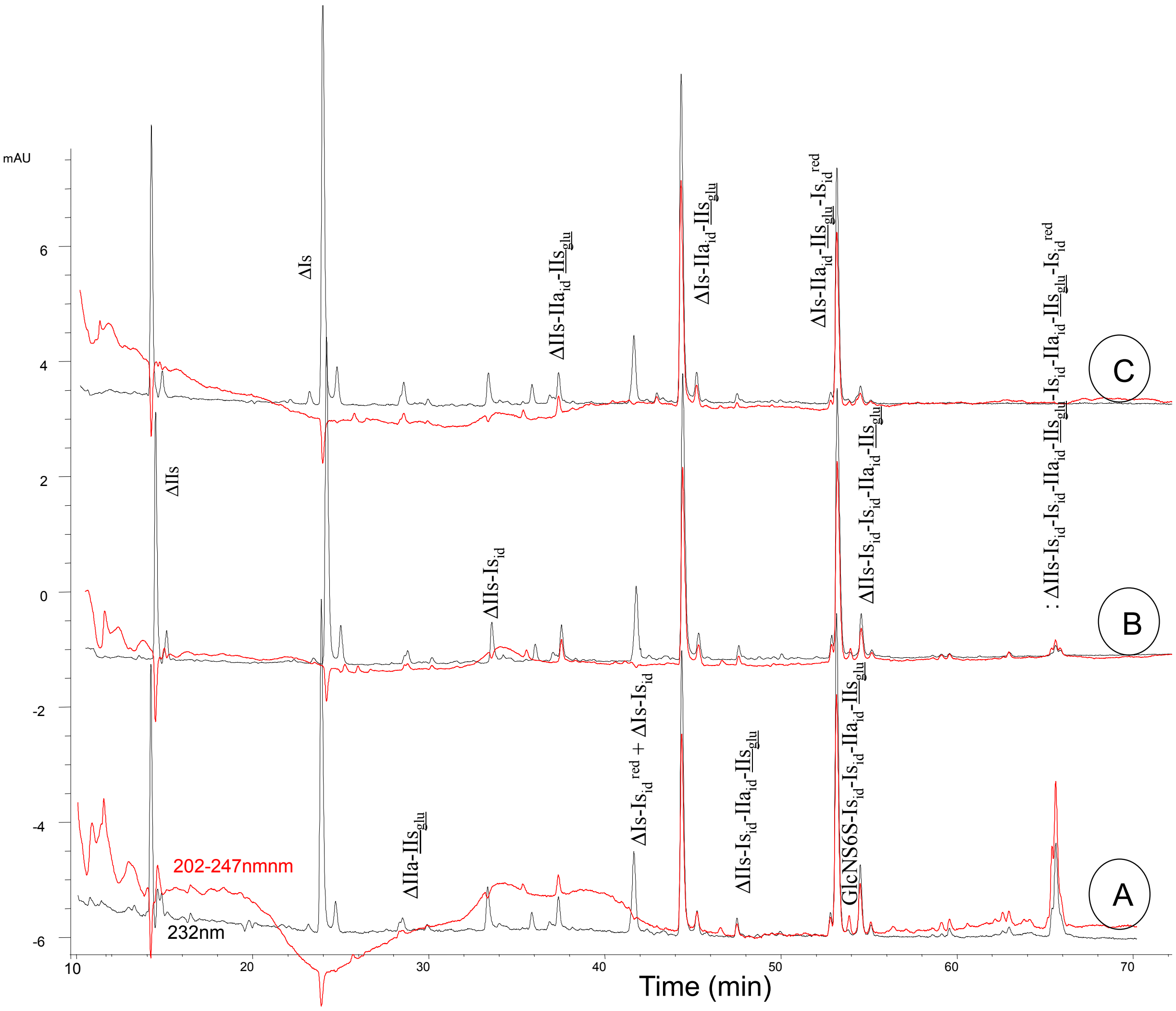

Figure S31. Sequencing of octadecasaccharide **6**, chromatographic follow-up on AS11 of the depolymerisation of octadecasaccharide6 by heparinase I.

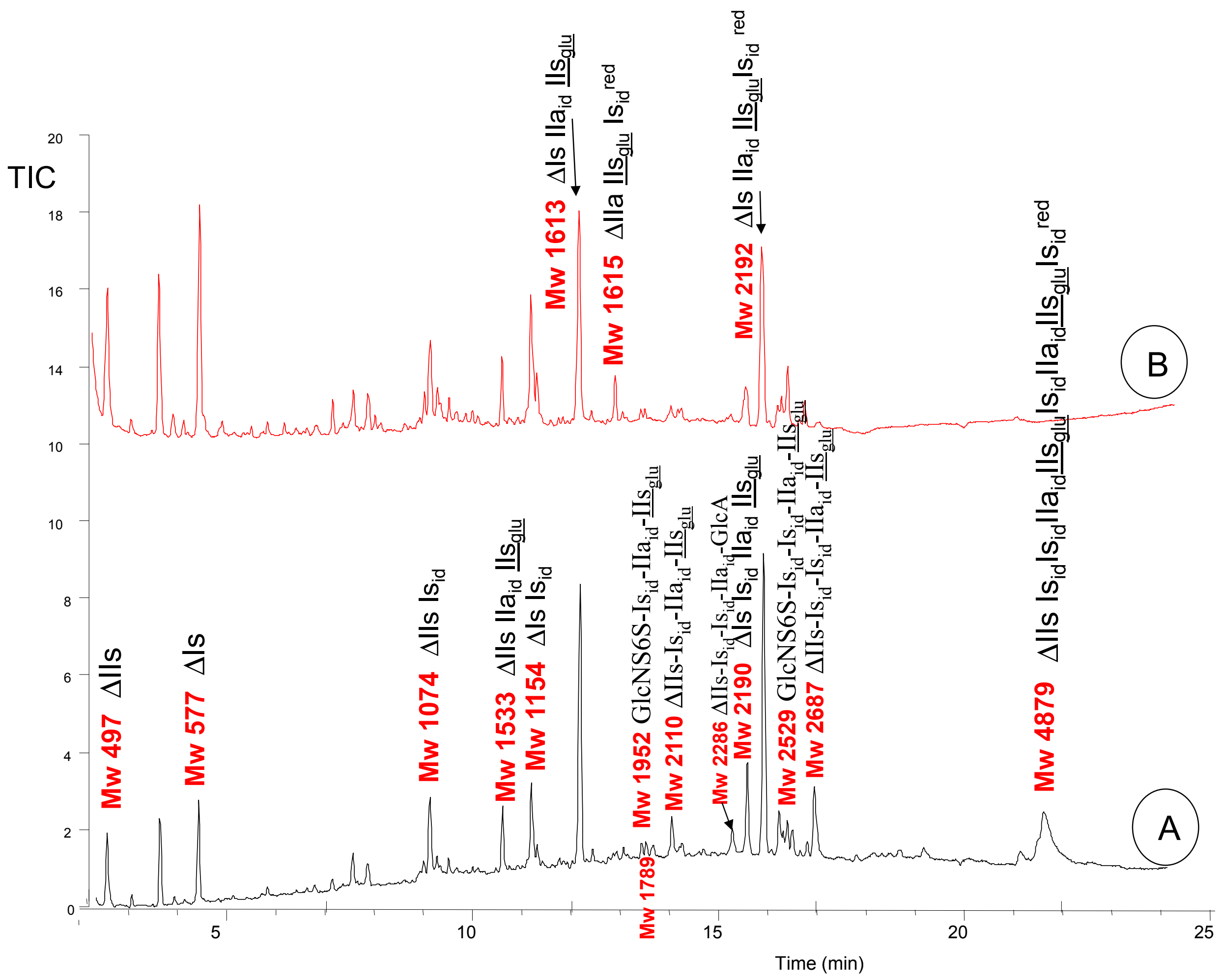

Figure S32. Sequencing of octadecasaccharide **6**, LC/MS follow-up of the depolymerisation of octadecasaccharide **6** by heparinase I.

NMR characterization of octadecasaccharides

Octadecasaccharide **2**

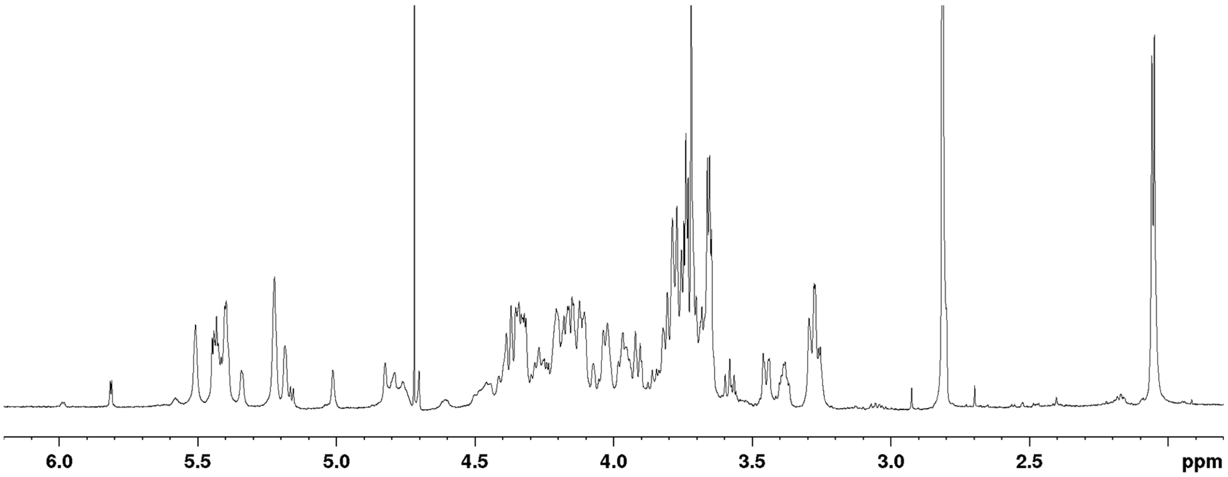

Figure S33. ^{1}H-NMR spectrum of octadecasaccharide **2** (D_2O, 30 °C, 600 MHz).

Table S1. Proton and carbon chemical shifts for the octadecasaccharide **2**: ΔIIa-$\underline{IIs}_{glu}$-Is_{id}-IIa_{id}-$\underline{IIs}_{glu}$-Is_{id}-Is_{id}-Is_{id}-Is_{id}.

	ΔIIa		IIs_{glu}		Is_{id}	
	ΔU	$A_{Nac,6S}$	G	$A_{NS,3S,6S}$	I_{2S}	$A_{NS,6S}$
1	5.16/102.1	5.42/98.0	4.61/102.2	5.51/97.2	5.18/100.6	5.34/96.6
2	3.82/71.5	3.95/54.6	3.39/74.6	3.45/57.8	4.33/77.8	3.26/59.0
3	4.24/68.0	3.81/70.4	3.70/77.5	4.37/77.3	4.17/71.4	3.66/70.7
4	5.81/108.8	3.86/79.2	3.82/77.6	3.97/74.1	4.15/77.1	3.79/76.7
5		4.05/70.1	3.78/78.3	4.16/70.7	4.80/71.2	3.97/70.4
6,6′		4.45/4.19		4.49/4.26		4.46/4.23
		67.2		67.1		67.4
CH_3		2.06/23.2				
	IIa_{id}		IIs_{glu}		Is_{id}	
	I	$A_{Nac,6S}$	G	$A_{NS,3S,6S}$	I_{2S}	$A_{NS,6S}$
1	5.01/103.1	5.39/98.0	4.60/102.2	5.51/97.2	5.18/100.6	5.40/98.0
2	3.79/69.8	3.93/55.0	3.38/74.6	3.45/57.8	4.33/77.8	3.28/59.1
3	4.12/68.9	3.78/70.6	3.70/77.5	4.37/77.3	4.17/71.4	3.67/70.7
4	4.07/75.8	3.73/78.4	3.80/77.6	3.97/74.1	4.15/77.1	3.78/76.6
5	4.79/69.7	4.02/70.3	3.76/78.2	4.16/70.7	4.80/71.2	4.03/70.4
6,6′		4.34/4.21		4.49/4.26		4.46/4.27
		67.4		67.1		67.3
CH_3		2.05/23.2				

	Is$_{id}$		Is$_{id}$		Is$_{id}$	
	I$_{2S}$	A$_{NS,6S}$	I$_{2S}$	A$_{NS,6S}$	I$_{2S}$	A$_{NS,6S}$
1	5.23/100.4	5.40/98.0	5.23/100.4	5.43/97.5	5.22/100.4	4.45/92.2
2	4.35/76.9	3.28/59.1	4.35/76.9	3.28/59.1	4.33/77.9	3.27/59.1
3	4.21/70.3	3.67/70.7	4.21/70.3	3.67/70.7	4.19/70.5	3.70/70.7
4	4.11/77.2	3.78/76.6	4.11/77.2	3.77/77.2	4.12/77.1	3.76/78.0
5	4.83/70.5	4.03/70.4	4.83/70.5	4.03/70.3	4.76/70.8	4.13/69.6
6,6′		4.40/4.28		4.40/4.28		4.38/4.31
		67.5		67.5		68.1
CH_3						

Note: Listed as H/C in ppm.

Octadecasaccharide **3**

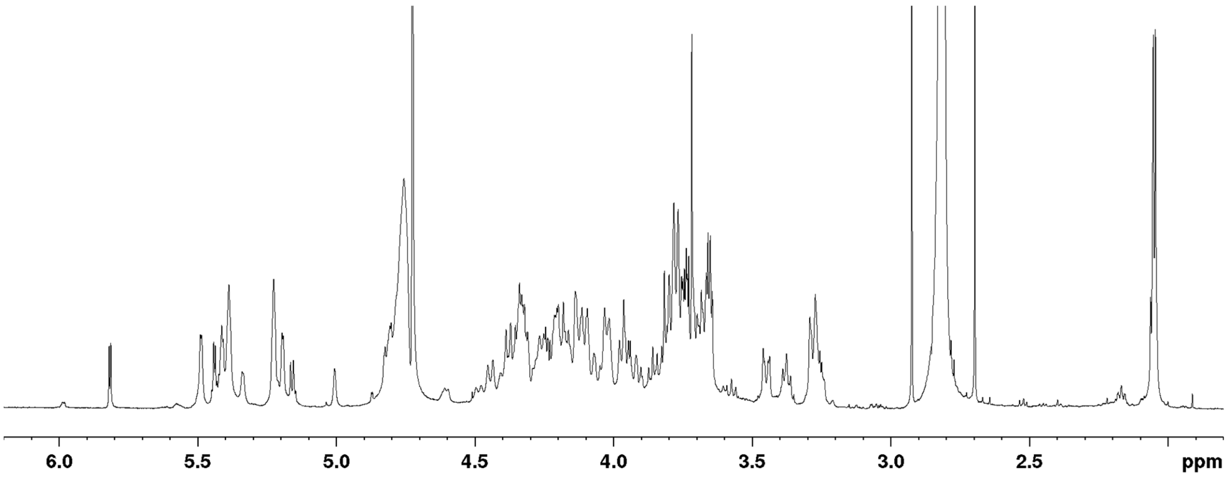

Figure S34. ^{1}H-NMR spectrum of octadecasaccharide **3** (D_2O, 30 °C, 600 MHz).

Table S2. Proton and carbon chemical shifts for the octadecasaccharide **3**: ΔIIa-$\underline{IIs_{glu}}$-Is$_{id}$-Is$_{id}$-IIa$_{id}$-$\underline{IIs_{glu}}$-Is$_{id}$-Is$_{id}$-Is$_{id}$.

	ΔIIa		IIs$_{glu}$		Is$_{id}$	
	ΔU	A$_{NAc,6S}$	G	A$_{NS,3S,6S}$	I$_{2S}$	A$_{NS,6S}$
1	5.16/102.1	5.41/97.8	4.60/102.2	5.49/97.3	5.19/100.5	5.39/98.0
2	3.81/71.5	3.95/54.5	3.38/74.6	3.45/57.8	4.33/77.6	3.28/59.1
3	4.24/68.0	3.80/70.4	3.69/77.5	4.37/77.4	4.18/71.1	3.67/70.7
4	5.82/108.8	3.86/79.0	3.80/77.6	3.97/74.1	4.13/77.0	3.78/76.9
5		4.04/70.1	3.76/78.2	4.15/70.8	4.81/71.0	3.97/70.3
6,6′		4.45/4.19		4.49/4.26		4.44/4.26
		67.3		67.1		67.3

CH_3		2.06/23.3				
	IS_{id}		IIa_{id}		IIS_{glu}	
	I_{2S}	$A_{NS,6S}$	I	$A_{NAc,6S}$	G	$A_{NS,3S,6S}$
1	5.23/100.3	5.34/96.5	5.01/103.1	5.39/98.0	4.60/102.2	5.49/97.3
2	4.35/76.7	3.25/59.1	3.79/69.6	3.93/55.0	3.38/74.6	3.45/57.8
3	4.21/70.1	3.66/70.7	4.12/68.8	3.77/70.6	3.69/77.5	4.37/77.4
4	4.10/77.1	3.77/77.1	4.07/75.7	3.73/78.3	3.78/77.8	3.97/74.1
5	4.83/70.3	3.97/70.3	4.79/69.7	4.02/70.3	3.76/78.2	4.16/70.8
6,6′		4.40/4.27		4.33/4.21		4.49/4.26
		67.5		67.4		67.1
CH3				2.05/23.3		
	IS_{id}		IS_{id}		IS_{id}	
	I_{2S}	$A_{NS,6S}$	I_{2S}	$A_{NS,6S}$	I_{2S}	$A_{NS,6S}$
1	5.19/100.5	5.39/98.0	5.23/100.3	5.41/97.6	5.22/100.3	4.44/92.2
2	4.33/77.6	3.28/59.1	4.35/76.7	3.28/59.1	4.33/77.6	3.26/59.1
3	4.18/71.1	3.67/70.7	4.21/70.1	3.67/70.7	4.19/70.3	3.70/70.7
4	4.13/77.0	3.78/76.9	4.10/77.1	3.77/76.9	4.11/77.0	3.76/78.0
5	4.81/71.0	3.97/70.3	4.83/70.3	4.04/70.1	4.77/70.6	4.13/69.6
6,6′		4.44/4.26		4.40/4.27		4.38/4.30
		67.3		67.5		68.0
CH3						

Note: Listed as H/C in ppm.

Octadecasaccharide **4**

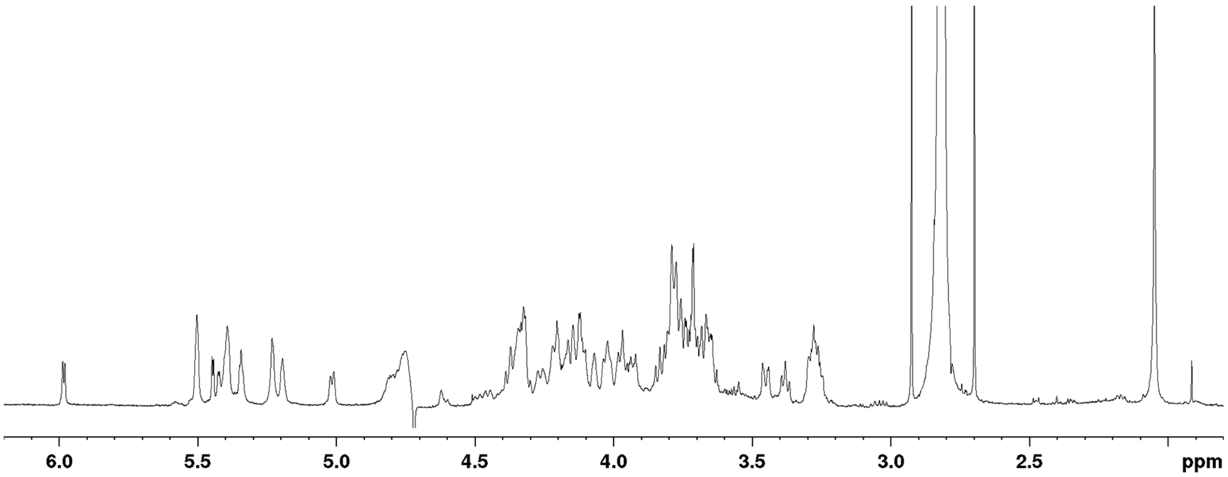

Figure S35. ^{1}H-NMR spectrum of octadecasaccharide **4** (D_2O, 30 °C, 600 MHz).

Table S3. Proton and carbon chemical shifts for the octadecasaccharide **4**: ΔIs-IIa_{id}-$\underline{IIs_{glu}}$-Is_{id}-Is_{id}-IIa_{id}-$\underline{IIs_{glu}}$-Is_{id}- Is_{id}.

	ΔIs		IIa_{id}		IIs_{glu}	
	ΔU	$A_{NS,6S}$	I	$A_{Nac,6S}$	G	$A_{NS,3S,6S}$
1	5.50/98.3	5.35/96.7	5.01/103.0	5.39/98.0	4.61/102.2	5.51/97.2
2	4.62/75.6	3.28/58.6	3.78/69.7	3.93/54.9	3.38/74.5	3.45/57.7
3	4.32/63.9	3.65/70.7	4.13/68.8	3.77/70.6	3.70/77.4	4.38/77.3
4	5.98/107.1	3.84/79.2	4.07/75.9	3.73/78.4	3.80/77.7	3.97/74.0
5		3.98/69.8	4.78/69.6	4.02/70.3	3.76/78.2	4.16/70.7
6,6′		4.36/4.21		4.33/4.21		4.49/4.26
		67.3		67.3		67.0
CH_3				2.05/23.2		
	Is_{id}		Is_{id}		IIa_{id}	
	I_{2S}	$A_{NS,6S}$	I_{2S}	$A_{NS,6S}$	I	$A_{Nac,6S}$
1	5.20/100.5	5.40/97.8	5.23/100.4	5.34/96.5	5.02/103.1	5.39/98.0
2	4.34/77.9	3.29/59.0	4.34/77.9	3.26/58.9	3.79/69.7	3.93/54.9
3	4.17/71.3	3.67/70.7	4.21/70.2	3.67/70.7	4.12/68.9	3.78/70.6
4	4.15/77.0	3.79/76.6	4.11/77.1	3.78/77.1	4.08/75.7	3.74/78.4
5	4.81/71.1	4.03/70.3	4.82/70.5	3.98/70.3	4.79/69.7	4.02/70.3
6,6′		4.45/4.27		4.41/4.24		4.34/4.22
		67.4		67.4		67.3
CH3						2.05/23.2
	IIs_{glu}		Is_{id}		Is_{id}	
	G	$A_{NS,3S,6S}$	I_{2S}	$A_{NS,6S}$	I_{2S}	$A_{NS,6S}$
1	4.61/102.2	5.51/97.2	5.20/100.5	5.43/97.5	5.23/100.4	4.45/92.1
2	3.38/74.5	3.45/57.7	4.34/77.9	3.29/59.0	4.33/77.4	3.27/59.0
3	3.70/77.4	4.38/77.3	4.18/71.3	3.67/70.7	4.20/70.4	3.71/70.6
4	3.80/77.7	3.97/74.0	4.15/77.0	3.79/76.6	4.12/77.1	3.76/78.2
5	3.76/78.2	4.16/70.7	4.81/71.1	4.03/70.3	4.77/70.7	4.13/69.5
6,6′		4.49/4.26		4.45/4.27		4.38/4.31
		67.0		67.4		68.0
CH3						

Note: Listed as H/C in ppm.

Octadecasaccharide **5**

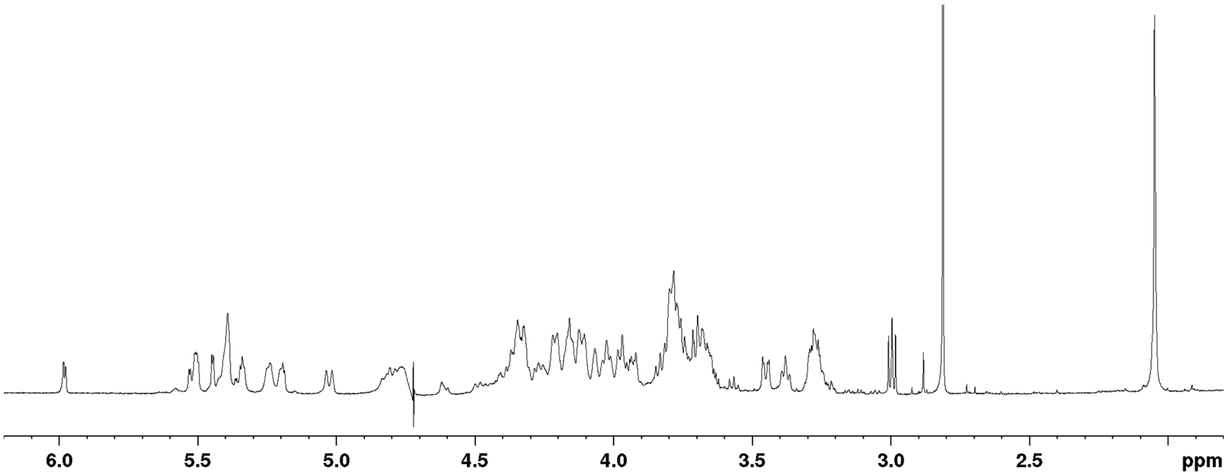

Figure S36. ^{1}H-NMR spectrum of octadecasaccharide **5** (D_2O, 30 °C, 600 MHz).

Table S4. Proton and carbon chemical shifts for the octadecasaccharide **5**: ΔIs-IIa_{id}-$\underline{IIs_{glu}}$-Is_{id}-Is_{id}-Is_{id}-IIa_{id}-$\underline{IIs_{glu}}$-Is_{id}.

	ΔIs		IIa_{id}		IIs_{glu}	
	ΔU	$A_{NS,6S}$	I	$A_{NAc,6S}$	G	$A_{NS,3S,6S}$
1	5.50/98.4	5.34/96.7	5.02/103.0	5.39/98.0	4.60/102.2	5.51/97.2
2	4.62/75.7	3.27/58.5	3.79/69.7	3.93/54.9	3.38/74.5	3.45/57.7
3	4.32/63.9	3.65/70.7	4.13/68.7	3.78/70.6	3.70/77.4	4.37/77.3
4	5.98/107.0	3.83/79.2	4.06/75.8	3.73/78.4	3.80/77.7	3.97/74.0
5		3.98/69.8	4.79/69.7	4.02/70.3	3.76/78.2	4.15/70.7
6,6′		4.36/4.21		4.33/4.21		4.49/4.26
		67.3		67.3		67.0
CH_3				2.05/23.2		
	Is_{id}		Is_{id}		Is_{id}	
	I_{2S}	$A_{NS,6S}$	I_{2S}	$A_{NS,6S}$	I_{2S}	$A_{NS,6S}$
1	5.20/100.4	5.40/97.8	5.25/100.3	5.41/97.7	5.24/100.3	5.33/96.6
2	4.33/77.9	3.29/59.0	4.35/76.7	3.28/59.0	4.33/77.9	3.25/58.9
3	4.17/71.4	3.67/70.7	4.21/70.1	3.68/70.6	4.20/70.2	3.68/70.7
4	4.15/77.0	3.80/76.6	4.10/77.2	3.78/76.7	4.11/77.1	3.78/77.0
5	4.81/71.2	4.03/70.3	4.84/70.5	4.03/70.3	4.82/70.6	3.97/70.3
6,6′		4.45/4.27		4.40/4.27		4.41/4.24
		67.4		67.4		67.4
CH3						
	IIa_{id}		IIs_{glu}		Is_{id}	
	I	$A_{NAc,6S}$	G	$A_{NS,3S,6S}$	I_{2S}	$A_{NS,6S}$
1	5.04/103.0	5.39/98.0	4.60/102.2	5.53/97.1	5.19/100.6	4.45/92.1

2	3.80/69.6	3.93/54.9	3.38/74.5	3.45/57.7	4.32/78.4	3.27/59.0
3	4.12/68.8	3.78/70.6	3.70/77.4	4.36/77.3	4.17/71.4	3.70/70.6
4	4.07/75.7	3.74/78.4	3.80/77.7	3.97/74.0	4.16/77.1	3.79/77.6
5	4.81/69.8	4.02/70.3	3.76/78.2	4.16/70.7	4.75/71.5	4.12/69.6
6,6′		4.34/4.22		4.49/4.26		4.42/4.29
		67.3		67.0		67.9
CH3		2.05/23.2				

Note: Listed as H/C in ppm.

Reference

1. Mourier, P.A.; Guichard, O.Y.; Herman, F.; Viskov, C. Isolation of a pure octadecasaccharide with antithrombin activity from an ultra-low-molecular-weight heparin. *Anal. Biochem*. **2014**, *453*, 7–15.
